# Impact of neonatal iron deficiency on hippocampal DNA methylation and gene transcription in a porcine biomedical model of cognitive development

**DOI:** 10.1186/s12864-016-3216-y

**Published:** 2016-11-03

**Authors:** Kyle M. Schachtschneider, Yingkai Liu, Laurie A. Rund, Ole Madsen, Rodney W. Johnson, Martien A. M. Groenen, Lawrence B. Schook

**Affiliations:** 1 Department of Animal Sciences, University of Illinois, 1201 W Gregory Drive, Urbana, IL 61801 USA; 2 Animal Breeding and Genomics Centre, Wageningen University, P.O. Box 338, Wageningen, 6700AH The Netherlands; 3 College of Animal Science and Technology, Sichuan Agricultural University, Wenjiang, Huimin Road #221, Chengdu, 610000 China; 4 Institute for Genomic Biology, University of Illinois, 1206 W Gregory Drive, Urbana, IL 61801 USA

**Keywords:** Neonatal iron deficiency, Porcine biomedical model, DNA methylation, Gene expression, Angiogenesis, Neurodevelopment, Hypoxia, RRBS, RNA-seq

## Abstract

**Background:**

Iron deficiency is a common childhood micronutrient deficiency that results in altered hippocampal function and cognitive disorders. However, little is known about the mechanisms through which neonatal iron deficiency results in long lasting alterations in hippocampal gene expression and function. DNA methylation is an epigenetic mark involved in gene regulation and altered by environmental factors. In this study, hippocampal DNA methylation and gene expression were assessed via reduced representation bisulfite sequencing and RNA-seq on samples from a previous study reporting reduced hippocampal-based learning and memory in a porcine biomedical model of neonatal iron deficiency.

**Results:**

In total 192 differentially expressed genes (DEGs) were identified between the iron deficient and control groups. GO term and pathway enrichment analysis identified DEGs associated with hypoxia, angiogenesis, increased blood brain barrier (BBB) permeability, and altered neurodevelopment and function. Of particular interest are genes previously implicated in cognitive deficits and behavioral disorders in humans and mice, including *HTR2A*, *HTR2C*, *PAK3*, *PRSS12*, and *NETO1*. Altered genome-wide DNA methylation was observed across 0.5 million CpG and 2.4 million non-CpG sites. In total 853 differentially methylated (DM) CpG and 99 DM non-CpG sites were identified between groups. Samples clustered by group when comparing DM non-CpG sites, suggesting high conservation of non-CpG methylation in response to neonatal environment. In total 12 DM sites were associated with 9 DEGs, including genes involved in angiogenesis, neurodevelopment, and neuronal function.

**Conclusions:**

Neonatal iron deficiency leads to altered hippocampal DNA methylation and gene regulation involved in hypoxia, angiogenesis, increased BBB permeability, and altered neurodevelopment and function. Together, these results provide new insights into the mechanisms through which neonatal iron deficiency results in long lasting reductions in cognitive development in humans.

**Electronic supplementary material:**

The online version of this article (doi:10.1186/s12864-016-3216-y) contains supplementary material, which is available to authorized users.

## Background

Iron is a micronutrient required for a number of important biological processes including neurological functions such as myelination and neurotransmission [1]. Iron deficiency is one of the most common micronutrient deficiencies in the world and disproportionately affects children under 5 years of age [2], resulting in altered brain function that can lead to cognitive and behavioral disorders [1]. While these symptoms can be reversed through iron supplementation in individuals suffering from iron deficiency during adolescence, the effects of neonatal iron deficiency are long-lasting and potentially permanent [3, 4]. This is likely due to the important developmental brain growth and maturation that takes place during the first few years of life, characterized by dendritic growth, synaptogenesis, and glial cell proliferation [5, 6].

One of the brain regions important for learning and memory functions, as well as emotional responses is the hippocampus [6]. Numerous studies have reported altered hippocampal gene expression in individuals with reduced learning and memory or behavioral changes [7–9], and the hippocampus is known to be highly vulnerable to early life environmental insults such as iron deficiency [10]. However, little is known about the cellular mechanisms responsible for long lasting alterations in hippocampal gene expression and function in response to early life iron deficiency.

Epigenetics is the study of changes in gene expression and phenotypes that occur without changes to the DNA sequence [11]. DNA methylation is an epigenetic mark that occurs predominantly at cytosine bases located 5’ adjacent to a guanine (CpG sites) throughout the genome, has a function in gene regulation, and can be altered in response to environmental factors [11]. In addition, cytosine methylation outside of CpG contexts (non-CpG sites) has been observed in embryonic stem cells, induced pluripotent stem cells, and oocytes of many mammalian species [12–14], as well as mammalian and avian neuronal tissue [15–17]. As DNA methylation is affected by environmental exposures and involved in gene regulation, it is expected that environmentally induced phenotypic changes can be explained in part by aberrant DNA methylation resulting in altered gene expression.

Recently neonatal pigs have emerged as ideal translational models for neurodevelopment studies due to a number of similarities they share with humans [18]. Both humans and pigs experience a similar neonatal brain growth spurt, and the gyral pattern and distribution of gray and white matter are similar in piglets and human infants [19]. Pigs can also undergo behavioral testing to assess cognitive development as early as 2 weeks of age [20]. In addition, as in humans, pigs are born with limited iron stores and are susceptible to iron deficiency without a proper iron source [21]. This, in combination with the similar distribution and function of DNA methylation in porcine and human tissues [22, 23] makes pigs ideal models for studying the epigenetic and gene regulatory patterns associated with environmentally induced reductions in cognitive development. Therefore, the aim of this study was to identify alterations in hippocampal DNA methylation and gene expression patterns partially responsible for the observed reduced hippocampal-based spatial learning and memory phenotype in a porcine model of neonatal iron deficiency [18]. The results presented here provide new insights into the epigenetic and biological mechanisms behind the long lasting reductions in cognitive development observed in humans suffering from neonatal iron deficiency.

## Methods

### Study aims and design

The aim of this study was to identify altered hippocampal DNA methylation and gene expression responsible for previously reported cognitive impairments in response to neonatal iron deficiency [18]. Complete details on animals, housing, study design, and tissue collection can be found in Rytych et al. [18]. Briefly, naturally farrowed piglets were weaned at postnatal day 2, at which point they were artificially reared in caging units and provided sow milk replacer that was adequate, mildly deficient, or severely deficient in iron (100, 25, or 10 mg iron/kg milk solids, respectively). Hippocampal tissue samples were obtained at 4 weeks of age after assessment in a hippocampal-dependent spatial T-maze task. DNA and RNA were isolated and used herein to assess DNA methylation and gene transcription patterns induced by neonatal iron deficiency. Only tissue samples from piglets provided milk replacer with adequate or severely deficient iron levels (referred to as control and iron deficient, respectively) were assessed.

### Study design and sample collection

Hippocampus samples used in this study were collected from 4 week old female Yorkshire piglets (four control, three iron deficient) from a previously published study [18]. Complete details on animals, housing, study design, and tissue collection can be found in Rytych et al. [18]. Briefly, naturally farrowed piglets were weaned at postnatal day 2, at which point they were artificially reared in caging units and provided sow milk replacer that was adequate, mildly deficient, or severely deficient in iron (100, 25, or 10 mg iron/kg milk solids, respectively). Hippocampal and prefrontal cortex tissue samples were obtained at 4 weeks of age after assessment in a hippocampal-dependent spatial T-maze task. DNA and RNA were isolated and used herein to assess DNA methylation and gene transcription patterns induced by neonatal iron deficiency. Only tissue samples from piglets provided milk replacer with adequate or severely deficient iron levels (referred to as control and iron deficient, respectively) were assessed. The samples were rinsed with PBS and stored at −80 °C until processing.

### DNA and RNA isolation

Isolation of DNA and RNA from frozen hippocampus was performed simultaneously using the AllPrep DNA/RNA Mini Kit (Qiagen, Valencia, CA, USA) following the manufacturer’s instructions. The DNA concentration was determined using a NanoDrop spectrophotometer and DNA quality was assessed by electrophoresis using a 1 % agarose gel. RNA concentrations were determined using a NanoDrop spectrophotometer and analyzed by an Agilent 2100 Bioanalyzer using an RNA Nano bioanalyzer chip to determine RNA integrity as well as the presence/absence of gDNA by the Carver High-Throughput DNA Sequencing and Genotyping Unit (HTS lab, University of Illinois, Urbana, IL, USA). Only RNA samples with a RNA integrity number (RIN) greater than 7 were used for sequencing.

### Reduced representation bisulfite sequencing and targeted control library preparation

Reduced representation bisulfite sequencing (RRBS) libraries were produced using 2 μg of high-quality DNA by the HTS lab following standard protocols as previously described [22]. RRBS libraries were produced using DNA fragments ranging from 30 to 160 bp and quantified using Qubit (Life Technologies, Carlsbad, CA, USA), diluting to a final concentration of 10 nM. RRBS libraries were further quantitated by qPCR on an ABI 1900. Production of targeted control libraries were produced following standard RRBS protocols minus the bisulfite treatment step as previously described [22], as this method has been shown to be more effective at targeting CpG rich regions than traditional SNP detection methods [22].

### RNA-seq library preparation

TruSeq Stranded RNA-seq libraries (TruSeq Stranded RNA Sample Preparation Kit, Illumina, San Diego, CA, USA) were produced by the HTS lab using 1 μg of high-quality RNA following standard protocols as previously described [22]. RNA-seq libraries underwent 10 cycles of PCR amplification using the Kapa HiFi polymerase (Kapa Biosystems, Woburn, MA, USA) to reduce PCR duplication. Finally, quantification of the resulting RNA-seq libraries was performed as described above.

### Illumina sequencing

Illumina sequencing was performed by the HTS lab on an Illumina HiSeq2500. Single-end 100 bp reads were produced for RRBS and targeted control libraries, while paired-end 100 bp reads were produced for RNA-seq libraries.

### RRBS data analysis

Illumina sequencing resulted in an average of 52.3 million raw reads per sample, ranging from 37.6 to 61.1 million (Additional file 1: Table S1). Adapters were trimmed from reads using Trim Galore v.0.3.3 [24], which also removes experimentally introduced cytosines and filters reads based on minimum quality score (20) and length (20 bp). Trimmed reads were aligned to an in silico converted reduced representation version (20 to 180 bp fragments) of the swine reference genome [25] produced using BS-seeker2 v.2.0.5 [26]. Alignments were performed using BS-seeker2 v.2.0.5, which utilizes Bowtie2 v.2.1.0 [27], by adjusting the alignment mode (local), seed length (20), maximum number of mismatches allowed in the seed alignment (1), and maximum number of mismatches/read (2). The final alignments had an average coverage of 23.84 with 1.31 % of the genome covered (Additional file 1: Table S2). The ratio of unmethylated to total reads for covered cytosines on the mitochondrial genome was calculated to determine the bisulfite conversion rate (99.2 %). The bs_seeker2-call_methylation.py script was used to identify the ratio of methylated/total uniquely aligned reads at each site (methylation levels). Reads aligning to each strand were combined, and the minimum coverage for a site to be utilized for analysis was 10x in all samples.

Differentially methylated (DM) sites were calculated using methylKit v.0.9.4 [28] after removing sites with high read coverage (upper 99.9^th^ percentile) in order to eliminate bias caused by PCR duplication. Sites were considered DM with a minimum methylation difference of 25 % and a *q*-value < 0.01, which are program defaults intended to help ensure identified DM sites have potential biological significance.

### Targeted control data analysis

Targeted control datasets were used to assess CpG and non-CpG sites for potential SNPs as previously described [22]. Illumina sequencing resulted in an average of 15.5 million raw reads per sample, ranging from 12.6 to 18.5 million (Additional file 1: Table S1). Following trimming as described above, bowtie2 v.2.2.3 was used to perform alignments to the swine reference genome by adjusting the alignment mode (--end-to-end), the –N option (1), and the –L option (20). GATK v.2.3-9-ge5ebf34 [29] was used to realign the uniquely aligned reads. The final alignments had an average coverage of 7.17 with 1.28 % of the genome covered (Additional file 1: Table S2). GATK v.3.3-0 was used to perform variant calling by adjusting the –stand_call_conf option (50), the –stand_emit_conf option (20), and the –dcov option (200). Read depths used to call SNPs ranged from 1/3 to 2 times the average coverage (minimum depth of 4 reads), and taking the mapping quality (minimum of 20) into account. These sites were removed from the RRBS dataset before analysis.

### RNA-seq expression analysis

Illumina sequencing resulted in an average of 35.3 million raw paired-end reads per sample, ranging from 29.7 to 42.8 million (Additional file 1: Table S1). Sequential trimming for adapter contamination and A-tails, as well as minimum quality score (20) and length (20 bp) was performed as described above. While a minimum length of 20 bp was used for paired reads, a minimum length of 35 bp was used for unpaired reads. Tophat v.2.2.10 [30] was utilized to perform alignment to the swine reference genome as previously described [22]. Reads with more alignments than the maximum allowable number (20) were filtered out using the –M option. The remaining reads were aligned to the Ensembl swine reference transcriptome (-G) followed by alignment to the genome by adjusting the –read-realign-edit-dist option (0), the –mate-inner-dist option (120), the –mate-std-dev option (260), and by indicating the method used to perform the stranded library preparation (fr-firststrand). Cufflinks v.2.2.1 [31] was used to perform differential gene expression analysis as previously described [32]. Transcripts were assembled using cufflinks using the fr-firststrand option, after which transcripts from all samples were merged with the reference transcripts using Cuffmerge. Gene expression was pre-computed using Cuffquant by including the –u and fr-firststrand options. Finally, differential expression analysis was performed using Cuffdiff by including the –u and fr-firstrand options, and genes with a *q* < 0.05 were considered differentially expressed genes (DEGs).

### GO term and pathways analysis

GO terms and pathways enriched for DEGs were determined using DAVID v6.7 [33], with all genes as a background. Three domains (cellular component, molecular function, and biological process) were used to perform the GO term analysis, while KEGG [34], PANTHER [35], and REACTOME [36] databases were used for pathway analysis. The Benjamini-Hochberg method was used for multiple testing correction of *P*-values, with GO terms and pathways with a *q* < 0.05 reported as enriched.

### Statistical analysis

R v.3.1.2 [37] was utilized for statistical analysis. The Shapiro-Wilk normality test was used to assess data normality, and equality of variance was assessed using an F-test. The Student’s *t*-test was used to determine statistical significance between normally distributed groups with equal variance, while groups with unequal variance were tested using a Welch two sample *t*-test. Non-normally distributed data was tested using the Wilcoxon signed-rank test. Correlation analysis was performed using the Pearson correlation coefficient, and ANOSIM was performed using Bray-Curtis dissimilarities. Enrichment analysis was performed using a Fisher’s exact test. Significance for all tests was based on a *p*-value < 0.05, with the exception of tests requiring multiple testing corrections. Sites were considered DM with a *q*-value < 0.01, genes were considered DEGs with a *q*-value < 0.05, and GO terms and pathways were considered enriched with a *q*-value < 0.05.

## Results

### Iron deficiency and reduced cognition phenotypes

The aim of this study was to identify altered hippocampal DNA methylation and gene expression responsible for previously reported cognitive impairments in response to neonatal iron deficiency [18]. In that study, significant reductions in iron levels were observed in the hippocampus of iron deficient pigs, compared to a similar but non-significant reduction in the prefrontal cortex. In addition, increased expression of the transferrin receptor *TFRC* was observed in the hippocampus and prefrontal cortex of iron deficient pigs, although the difference was only significant in the prefrontal cortex. These results led to the conclusion that increased *TFRC* expression in the prefrontal cortex allows for the maintenance of higher prefrontal cortex iron concentrations, while the hippocampus was unable to adapt to properly protect itself from iron deficiency. The same reductions in iron concentration and increases in *TFRC* expression were observed in the pigs selected for this study (Additional file 1: Figure S1).

The previous study also assessed the effects of iron deficiency on hippocampal-based spatial learning and memory using a maze with visual cues indicating the location of the reward [18]. In that study, the iron deficient pigs made fewer correct choices, took longer to make a choice, and moved a greater distance in the maze, all of which indicate that iron deficiency resulted in reduced cognitive performance. These same trends in hippocampal-based spatial learning and memory were observed in the iron deficient and control pigs used in this study (Additional file 1: Figure S1). While not significant due to the lower sample size, the results clearly demonstrate that the pigs selected for this study are representative of their respective groups in terms of iron deficiency and cognitive functioning.

### Differential hippocampal gene expression by RNA-seq in a neonatal iron deficiency model of cognitive development

#### Evidence for hypoxia-induced angiogenesis and blood brain barrier permeability

**Fig. 1:**
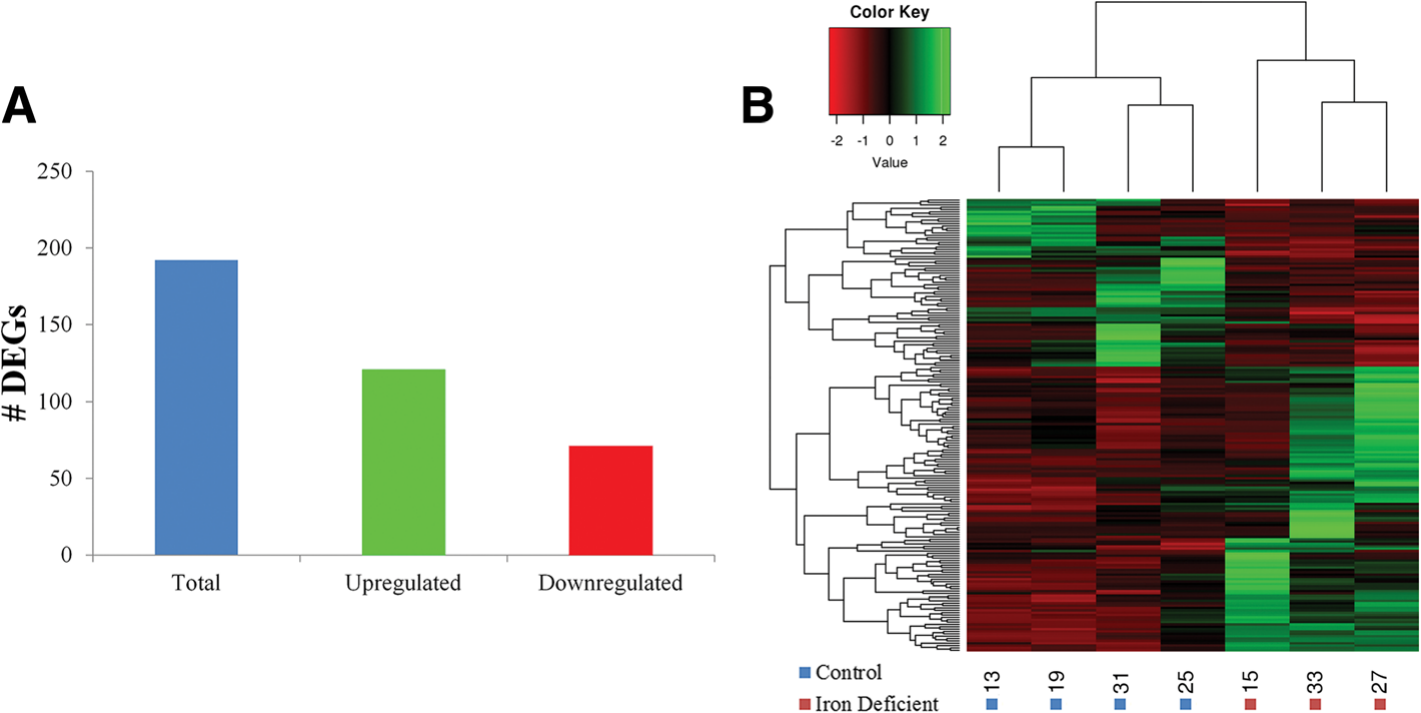
Visualization of DEGs. **a** Total number of DEGs, including those up- and down-regulated in the iron deficient compared to the control group. **b** Heatmap of the normalized expression level of the 192 DEGs for each sample, represented as z-scores. Dendrograms represent relationships between samples (*columns*) and genes (*rows*) based on complete linkage clustering

**Fig. 2:**
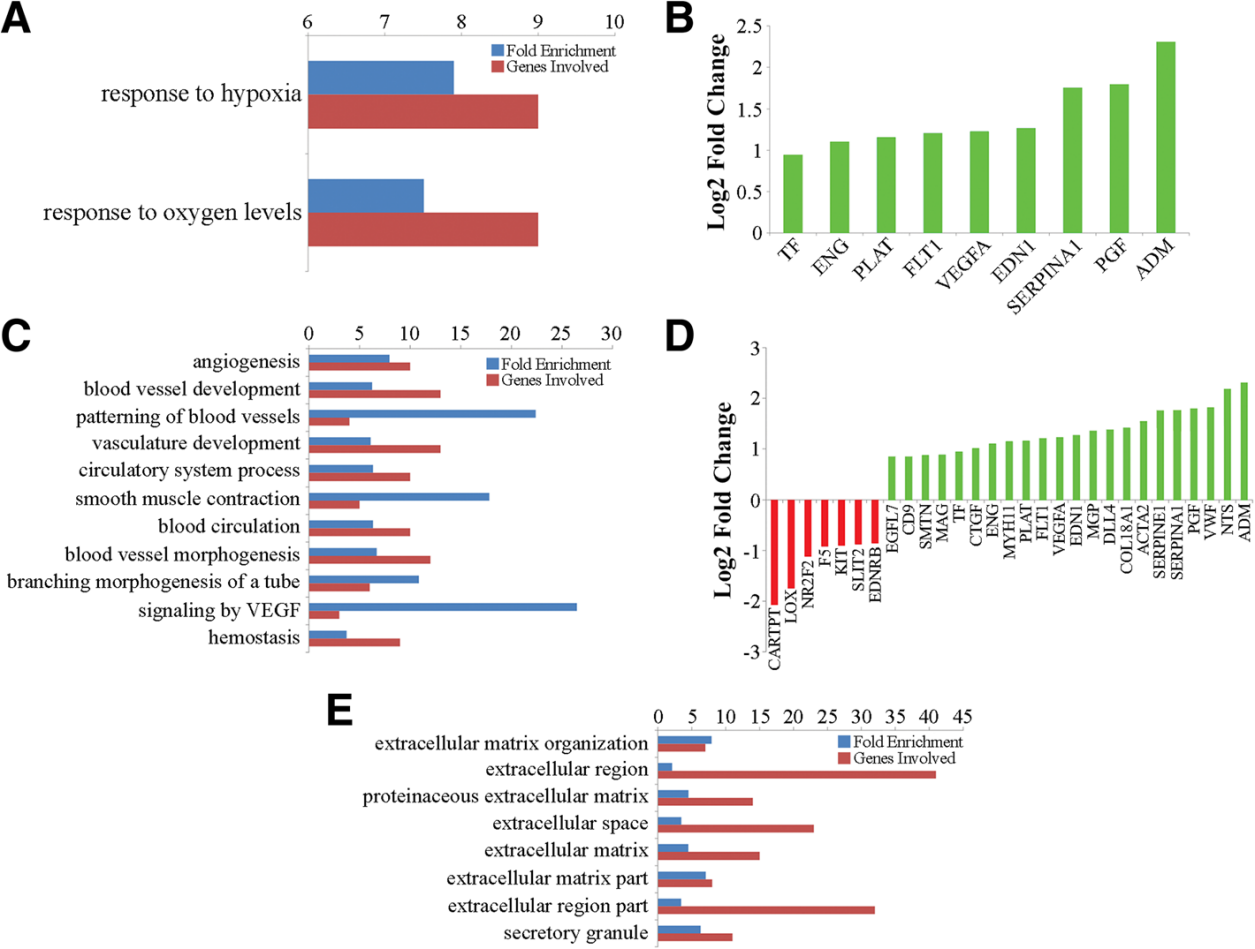
GO terms and pathways enriched for DEGs in the iron deficient group. **a**. GO terms related to response to hypoxia that were enriched for DEGs up-regulated in the iron deficient group. **b**. DEGs associated with enriched GO terms related to response to hypoxia. **c**. GO terms and pathways related to angiogenesis that were enriched for DEGs up-regulated in the iron deficient group. **d**. DEGs associated with enriched GO terms and pathways related to angiogenesis. **e**. GO terms and pathways related to genes located within the extracellular matrix that were enriched for DEGs up-regulated in the iron deficient group

A total of 192 differentially expressed genes (DEGs) were detected between the iron deficient and control groups, with 121 up- and 71 down-regulated in the iron deficient group (Fig. 1a, b, Additional file 1: Tables S3, S4). Log2 fold change differences ranged from 0.67 to 4.22 for up-regulated genes, and −0.69 to −4.03 for down-regulated genes (Additional file 1: Tables S3, S4). Cluster analysis based on the relative expression of the 192 DEGs resulted in samples clustering by group (Fig. 1b), suggesting a reproducible effect of iron deficiency on hippocampal gene expression. GO term and pathway enrichment analysis resulted in 37 GO terms and 5 pathways significantly enriched for DEGs, many of which are involved in response to hypoxia and angiogenesis, in addition to neurological system processes (Additional file 1: Table S5). Of the enriched GO terms and pathways, 28 and 3 were also enriched for DEGs up-regulated in the iron deficient group, respectively, including those related to response to hypoxia, angiogenesis, and genes located within the extracellular matrix (Fig. 2a, c, and e, Additional file 1: Table S6). In this study, all DEGs associated with response to hypoxia were up-regulated in the iron deficient group (Fig. 2b), including genes known to be up-regulated in hypoxic conditions such as *TF* [38], *PLAT* [39], *ENG* [40], *FLT1* [41], and *VEGFA* [42]. The majority of DEGs associated with angiogenesis related terms and pathways were also up-regulated in the iron deficient group (Fig. 2c), including a number of factors that are active during angiogenesis, such as *ENPP2* [43], *EGFL7* [44], *CD9* [45], *ENG* [46], *FLT1* [47], *VEGFA* [48], and *ADM* (Fig. 2d) [49].

In addition, increased *VEGFA* and *FLT1* expression is known to induce hyperpermeability of the blood brain barrier (BBB) [50]. *VWF* is another gene whose expression is associated with increased BBB permeability and was observed at higher levels in the iron deficient group (1.81 log2 fold change, Additional file 1: Table S3) [51]. Finally, increased expression of the vasoconstrictor receptor *EDNRB* is associated with vasoconstriction of cerebral arteries in rodent models of ischemic stroke [52]. Decreased expression of *EDNRB* was observed in the iron deficient group (−0.86 log2 fold change, Additional file 1: Table S4), suggesting vasodilation of hippocampal arteries. Together, these results suggest reduced hippocampal iron levels induced hypoxic conditions, resulting in hypoxia-induced angiogenesis and BBB permeability involved in the observed reduction in hippocampal-based spatial learning and memory.

#### Evidence for altered hippocampal neurodevelopment and function

**Fig. 3:**
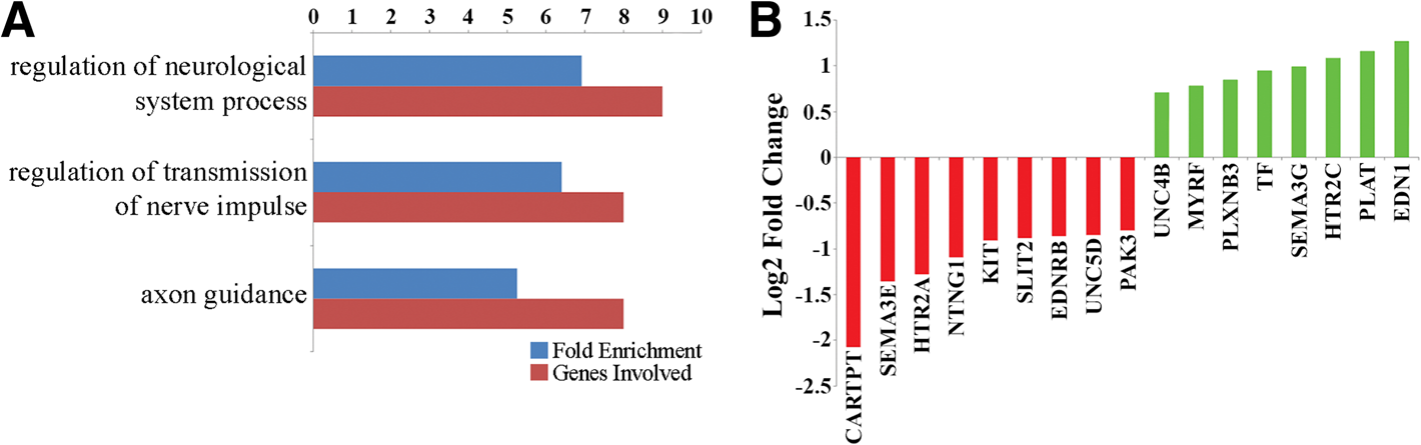
Enriched GO terms and pathways related to neurological functions in the iron deficient group. **a** GO terms and pathways enriched for DEGs related to neurodevelopment and function. **b** DEGs associated with enriched GO terms and pathways related to neurodevelopment and function

A number of enriched GO terms and pathways were related to neurological development and function (Fig. 3a, Additional file 1: Table S5), including the hsa04360:Axon guidance pathway. This pathway was enriched for all DEGs and down-regulated DEGs in the iron deficient group (Fig. 3a, Additional file 1: Table S5, S7), suggesting altered neurodevelopment in the iron deficient hippocampus. Many of the down-regulated DEGs are involved in neurodevelopment by promoting neurite or axon outgrowth, including *SEMA3E* [53], *NTNG1* [54], and *CARTPT*, which is also involved in stress responses and sensory processing (Fig. 3b) [55]. Additional down-regulated DEGs such as *SLIT2* [56] and *UNC5D* [57] have been shown to function as repulsive neuronal and axon guidance molecules (Fig. 3b). In addition, a number of up-regulated DEGs also promote neurodevelopment, including *UNC5B* [58], *SEMA3G* [59], *PLXNB3* [60] and *PLAT* [61] (Fig. 3b). The simultaneous up- and down-regulation of genes promoting neurodevelopment, including guidance cues (NTNG1, SLIT2, SEMA3E, and SEMA3G) and growth cone receptors (UNC5B, UNC5D, and PLXNB3) in the hsa04360:Axon guidance pathway, suggests iron deficiency resulted in altered hippocampal neurodevelopment.

Many of the DEGs associated with the enriched terms and pathways were also related to neuronal function, including two serotonin receptors, *HTR2A* and *HTR2C* (Fig. 3b). Serotonin is a neurotransmitter involved in many behavioral and functional processes including pain, emotion, and learning and memory [62]. In this study, both reduced *HTR2A* and increased *HTR2C* expression was observed in the iron deficient group (Fig. 3b), suggesting opposing downstream functions of these genes in response to binding by serotonin. Other down-regulated DEGs related to neuronal function include *PAK3* (Fig. 3b), which is involved in the formation and plasticity of synapses [63], *PRSS12* (−0.93 log2 fold change, Additional file 1: Table S4), which plays an essential role in maintaining neuronal plasticity in hippocampal neurons [64], and *NETO1* (−1.04 log2 fold change, Additional file 1: Table S4), a subunit of endogenous synaptic kainate receptors (KARs) required for synaptic plasticity and learning [65].

### Differential hippocampal DNA methylation by RRBS in a neonatal iron deficiency model of cognitive development

#### CpG and non-CpG site validation

As CpG sites mutate at a high rate due to the deamination of 5-methylcytosine to thymidine [66], which can lead to incorrect methylation calls, these sites were tested for the presence of SNPs using targeted control libraries as previously described [22]. Of the 1,145,787 high confidence CpG sites (covered by at least 10 reads in at least one sample), an average of 721,861 (63.00 %) were tested for the presence of SNPs in each individual, resulting in 19,597 (average of 8005/individual) detected SNPs that were removed before further analysis. In addition, of the 5,556,880 high confidence non-CpG sites, an average of 3,858,483 (69.44 %) were tested for the presence of SNPs in each sample, resulting in 15,179 (average of 6394/individual) detected SNPs that were removed from further analysis. In addition, 3,879,042 (69.81 %) high confidence non-CpG sites were tested for the presence of a guanine in the 3’ adjacent base that would result in the presence of a CpG site. A total of 12,532 (average of 6482/individual) non-CpG sites were converted to CpG sites, and were removed before further analysis. Furthermore, the average methylation level of converted CpG sites was higher in individuals found to contain the SNP (38.79 %) than individuals without the variant (3.89 %), further validating the use of targeted control libraries for SNP detection in RRBS studies.

**Fig. 4:**
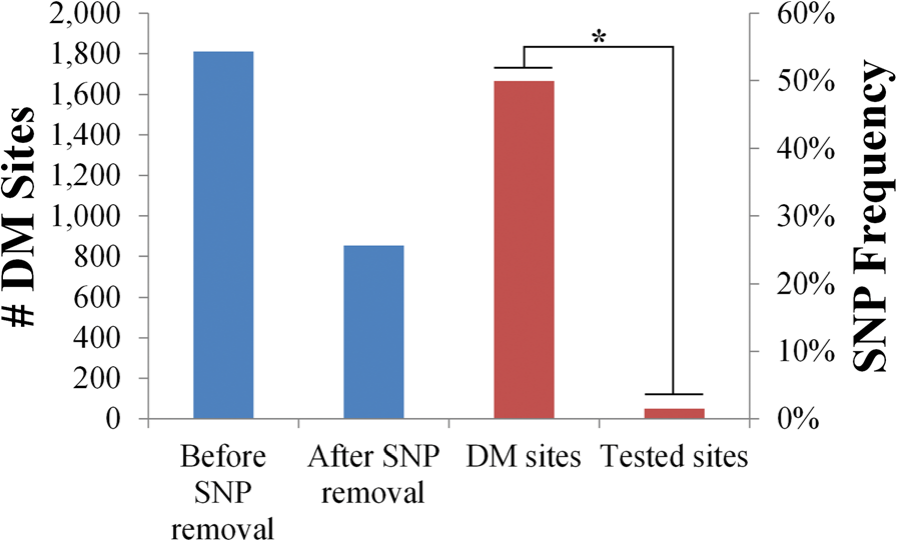
Influence of SNPs on differential methylation analysis. The number of DM CpG sites detected before and after SNP removal are displayed in *blue*, while the proportion of SNPs at DM CpG sites and all covered CpG sites are displayed in red. *denotes *P*-value < 1 x 10^−15^

In order to demonstrate the importance of SNP removal for studies involving differential methylation analysis, differentially methylated (DM) CpG sites were assessed between the control and iron deficient groups both before and after removal of SNP sites. A total of 853 DM CpG sites were detected after SNP removal, compared to 1810 before SNP removal (Fig. 4). Of the 957 additional DM CpG sites detected before SNP removal, 904 were located at detected SNP sites, resulting in a significant overrepresentation of SNPs at DM CpG sites (*P* < 1 x 10^−15^; Fig. 4). Taken together, these results further validate the use of targeted control libraries for SNP detection, while demonstrating the importance of SNP detection in studies assessing differential methylation.

#### Differential DNA methylation

**Fig. 5:**
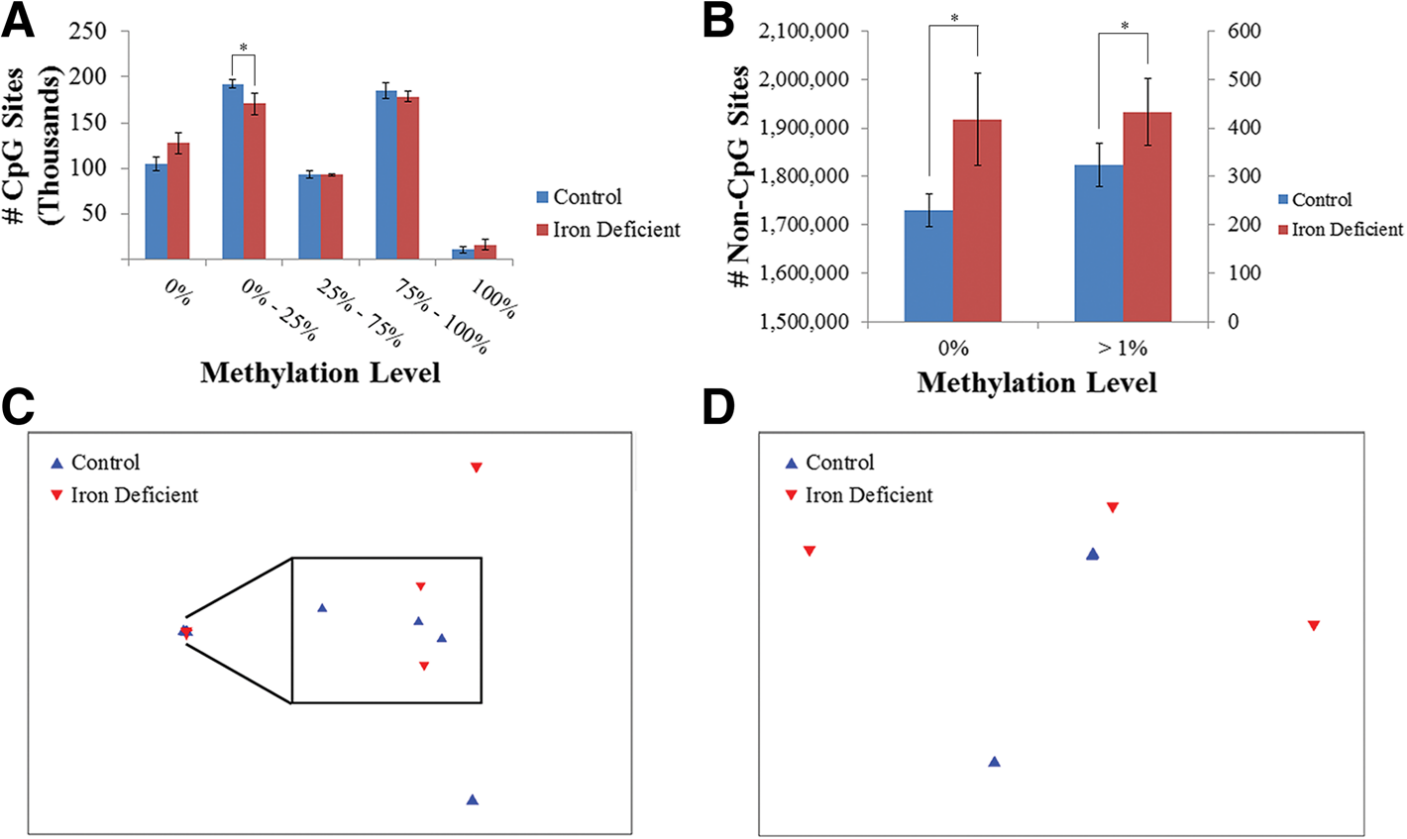
Distribution and similarity of hippocampal DNA methylation patterns. Distribution of **a** CpG and **b** non-CpG site methylation in the iron deficient and control samples. Multidimensional scaling plot of Bray-Curtis dissimilarities between samples for **c** all CpG sites and **d** all non-CpG sites. *denotes *p*-value < 0.05

A total of 584,669 CpG and 2,403,462 non-CpG sites were covered by all samples and used for comparative analysis. Significant differences in global methylation patterns were detected between groups, with a lower proportion of hypomethylated CpG sites (>0 % - < 25 %) observed in the iron deficient compared to the control group (*P* = 0.017; Fig. 5a). The vast majority of non-CpG sites were unmethylated in all samples (average of 75.88 %), indicating little non-CpG methylation present in the porcine neonatal hippocampus. Comparing non-CpG methylation levels revealed a higher proportion of unmethylated (0 %) and methylated (>1 %) non-CpG sites in the iron deficient compared to control group (*P* < 0.05; Fig. 5b). Globally samples were more similar in their CpG than non-CpG methylation patterns (average Pearson’s correlation coefficient of 0.98 and 0.81, respectively; *P* < 1 x 10^−15^; Additional file 1: Figure S2, S3), suggesting that genome-wide CpG methylation patterns are more conserved than non-CpG methylation patterns. Additionally, samples were not found to be more similar within groups than across groups when assessing methylation patterns at all CpG and non-CpG sites (ANOSIM *R* = −0.185 and 0.241, *P* = 0.888 and 0.129, respectively; Fig. 5c, d). Together the results suggest that early life iron deficiency leads to global changes in DNA methylation composition; however, these differences are not large enough to differentiate samples by group. Therefore, methylation differences at individual sites were assessed to identify regions potentially responsible for the observed gene expression changes between groups.

**Fig. 6:**
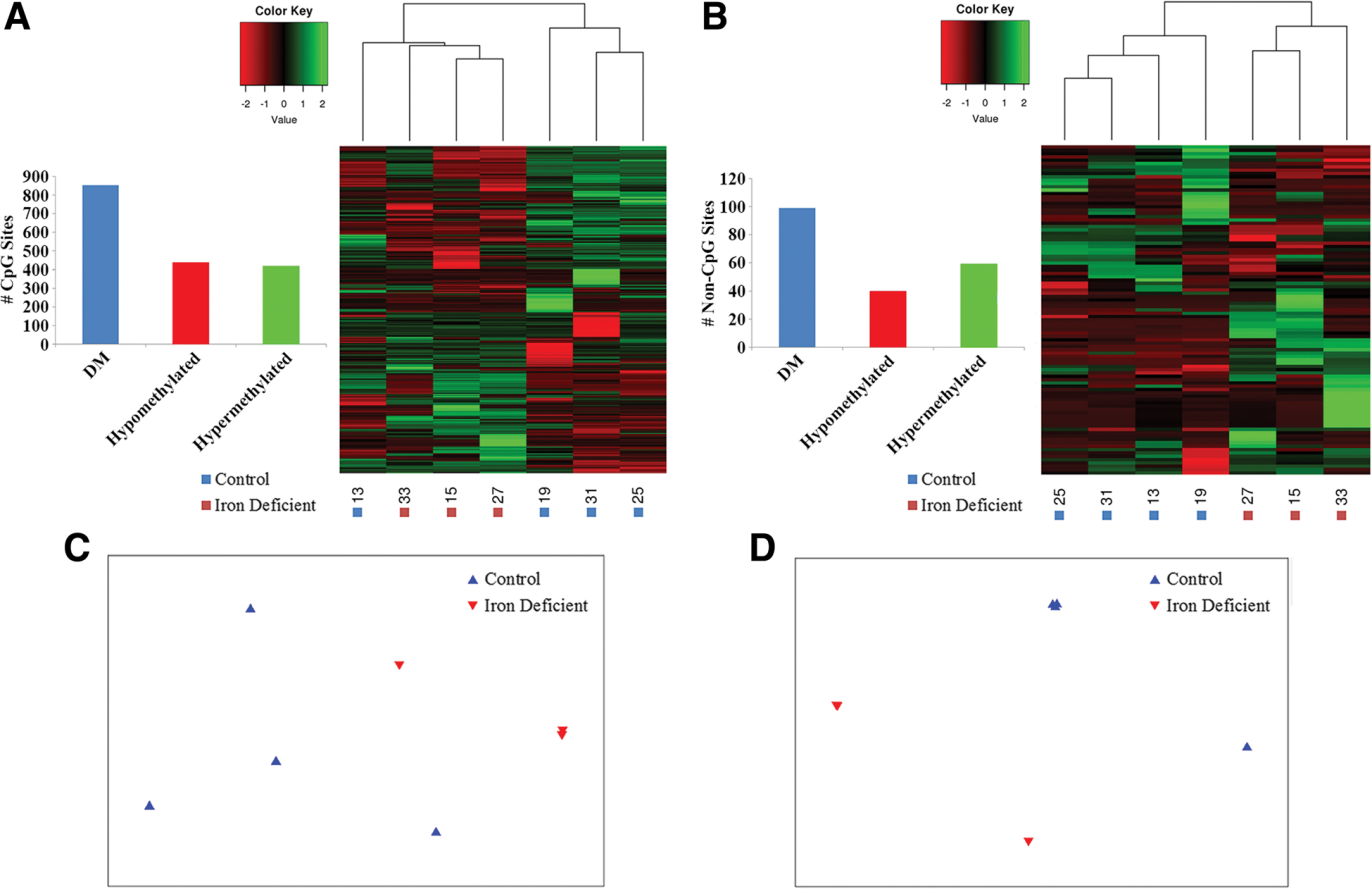
Distribution and similarity of DM sites across groups. Number of DM, hypomethylated, and hypermethylated **a** CpG and **b** non-CpG sites detected between the iron deficient and control group. Heatmap of the methylation level for **a** 853 DM CpG and **b** 99 DM non-CpG sites for each sample, represented as z-scores. Dendrograms represent relationships between samples based on complete linkage clustering. Multidimensional scaling plot of Bray-Curtis dissimilarities between samples for **c** DM CpG sites and **d** DM non-CpG sites

Differential methylation analysis revealed a total of 853 and 99 DM CpG and non-CpG sites between the iron deficient and control group, respectively. Similar numbers of DM CpG and non-CpG sites were observed to be hypo and hypermethylated in the iron deficient compared to the control group (Fig. 6a, b). Although samples were more highly correlated within groups than across when comparing DM CpG sites (average Pearson’s correlation coefficient 0.417 and 0.269, respectively), the result was not significant (ANOSIM *R* = 0.444, *P* = 0.077; Fig. 6c). This is due to the observed clustering of one control sample with the iron deficient samples when comparing DM CpG sites (Fig. 6a). However, samples were significantly more similar within groups than across when comparing DM non-CpG sites (average Pearson’s correlation coefficient 0.404 and 0.164, respectively, ANOSIM *R* = 0.5, *P* = 0.029; Fig. 6d), resulting in the clustering of samples by group (Fig. 6b).

**Fig. 7:**
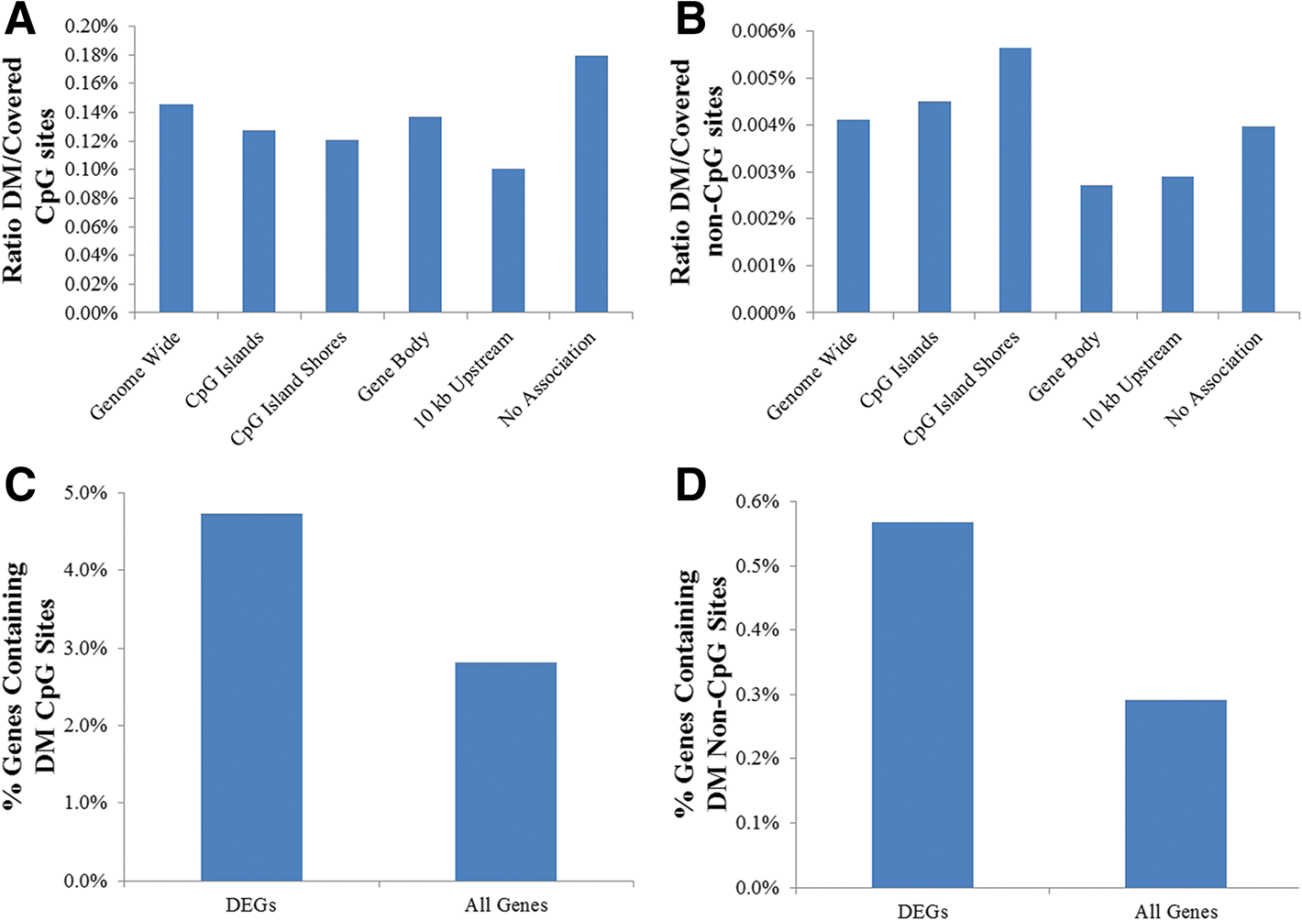
Distribution of DM sites in relation to genomic features. Ratio of DM to covered **a** CpG and **b** non-CpG sites in relation to genomic features. Distribution of **c** DM CpG and **d** DM non-CpG sites in DEG regions (including 10 kb upstream of TSS) compared to all genes

The genomic location of DM CpG and non-CpG sites in relation to genomic features was assessed by determining the ratio of DM to covered sites in CpG islands, shores, gene bodies, and up to 10 kb upstream of transcription start sites (TSS). The highest proportion of DM CpG sites had no association with known CpG islands, shores, or genes, with gene bodies containing slightly more DM CpG sites than CpG islands and shores, and the lowest proportion located 10 kb upstream of genes (Fig. 7a). In contrast, the highest proportion of DM non-CpG sites were located within CpG island shores, followed by CpG islands, with the lowest proportions located within gene bodies and 10 kb upstream of genes (Fig. 7b).

#### Differential methylation associated with differential expression

In order to determine if methylation changes were associated with changes in gene expression, DEGs were assessed for DM site enrichment compared to all other genes (including 10 kb upstream of TSS). While a higher proportion of DEGs were associated with DM CpG and non-CpG sites (4.73 and 0.57 %, respectively) compared to all other genes (2.82 and 0.29 %, respectively), these enrichments were not significant (*P* > 0.1; Fig. 7c, d). In total 11 DM CpG sites were located within or directly upstream of 8 DEGs (Table 1). It is important to note that while DNA methylation in general is negatively correlated with expression in the brain [17], recent studies suggest that DNA methylation can also lead to activation of transcription through various interactions with transcription factors [67]. Therefore, this study reported all DM sites associated with DEGs, regardless of the directional change in expression and methylation level.

**Table 1:** DM sites associated with DEGs

Gene	Chr	Start	Stop	Strand	Control expression (FPKM)	Iron deficient expression (FPKM)	Log2 fold change	CpG site	% Methylation difference
*AK5*	6	125,964,963	126,223,426	−	220.095	108.314	−1.02291	126,222,882	27.13 %
*COL18A1*	13	218,086,392	218,088,850	+	6.0639	16.1761	1.41555	218,086,634	46.84 %
*ENSSSCG00000010493*	14	116,376,967	116,425,960	−	0.787778	2.73519	1.79578	116,377,431	25.47 %
*SV2B*	7	93,628,494	93,828,585	−	61.2676	31.3412	−0.967065	93,828,414	29.24 %
*UNC5B*	14	80,010,893	80,098,908	+	7.68495	12.5631	0.709085	80,060,756	34.05 %
*VWF*	5	67,005,340	67,078,256	+	2.61282	9.17948	1.81281	67,050,813	−39.15 %
*HS6ST2*	X	124,748,953	125,078,245	−	21.3711	8.641	−1.30639	125,076,126	25.18 %
*HTR2C*	X	108,566,657	108,871,722	+	3.18817	6.73597	1.07916	108,591,537	37.11 %
*HTR2C*	X	108,566,657	108,871,722	+	3.18817	6.73597	1.07916	108,591,463	−35.23 %
*HTR2C*	X	108,566,657	108,871,722	+	3.18817	6.73597	1.07916	108,564,261	−25.29 %
*HTR2C*	X	108,566,657	108,871,722	+	3.18817	6.73597	1.07916	08,564,264	−37.58 %
Gene	Chr	Start	Stop	Strand	Control expression (FPKM)	Iron deficient expression (FPKM)	Log2 fold change	Non-CpG site	% Methylation difference
*MICALL2*	3	1,066,675	1,085,568	−	2.73823	6.46233	1.23881	1,084,014	44.53 %

The first 11 lines represent DM CpG sites associated with DEGs, while the last line represents a single DM non-CpG site associated with a DEG. % methylation difference is the observed change in methylation in the deficient group relative to the control group. Log2 fold changes are the observed change in expression in the deficient group relative to the control group. Expression levels are represented as fragments per kilobase of transcript per million fragments mapped (FPKM)

The majority the DEGs associated with DM sites are involved in either angiogenesis, neurodevelopment, or neuronal function, including the previously mentioned *VWF*, *UNC5B*, and *HTR2C* (Table 1). The observed increased *VWF* expression was associated with hypomethylation of an intragenic CpG site located between exons 11 and 12 in the iron deficient group (−39.15 %), while increased *UNC5B* expression was associated with hypermethylation of an intragenic CpG site located between exons 1 and 2 (34.05 %). Neither site was associated with a CpG island or shore. In addition, the observed increased *HTR2C* expression was associated with hypomethylation of 2 CpG sites located between 2390 and 2400 bp upstream of the TSS in the iron deficient group (Table 1), suggesting the presence of a promoter in this region. In addition, hyper- (37.11 %) and hypomethylation (−35.23 %) was observed at 2 intragenic CpG sites located between exons 1 and 2. Another DEG associated with differential methylation was *SV2B*, a synaptic vesicle protein involved in the regulation of Ca^++^ dependent synaptic neurotransmitter release in the hippocampus [68]. In this study, reduced expression (−0.97 log2 fold change) was observed in the iron deficient group associated with hypermethylation (29.24 %) of an intragenic CpG site located less than 200 bp from the TSS in exon 1 (Table 1).

In addition to the DM CpG sites, 1 DM non-CpG site was associated with *MICALL2* (Table 1), an effector protein of *RAB13* that inhibits neurite outgrowth [69]. *MICALL2* was expressed at a 1.24 log2 fold higher level in the iron deficient compared to the control group, suggesting an increase in hippocampal neurite outgrowth inhibition in this group. The increased expression was associated with hypermethylation (44.53 %) of an intragenic non-CpG site (CpC site) less than 1600 bp downstream of the TSS. In addition, this site is located within a CpG island shore separating 2 CpG islands, one of which overlaps the TSS.

## Discussion

This study sought to assess differences in hippocampal DNA methlyation and gene expression between control piglets and those with reduced hippocampal-based learning and memory as a result of neonatal iron deficiency. One of the detected DEGs of interest encodes the plasma glycoprotein transferrin (*TF*), which binds iron and transports it through the blood and across cell membranes by binding to its receptor *TFR* [70]. *TF* plays an important role in transporting iron to many brain regions including the hippocampus, where it is needed for enzyme activity, oxygen delivery, and neurotransmitter synthesis [70]. Increased hippocampal *TF* expression is observed in rats fed iron deficient diets [70], and was also observed in the iron deficient pigs. This, combined with the reduced hippocampal iron levels in the iron deficient pigs, provides evidence for the iron deficient status of the hippocampus.

Iron is essential for oxygen delivery, and the brain has the highest rate of oxygen consumption of any organ [71]. Reduced iron levels have been shown to cause hypoxia in the brain [72], resulting in a reduction in hippocampal gray and white matter volumes, as well as cognitive and motor difficulties in mice [73]. The enrichment of response to hypoxia related GO terms and pathways, in addition to reduced hippocampal iron levels, suggests hypoxic conditions were present in the iron deficient hippocampus. Hypoxic conditions in the brain are one of the primary regulators of angiogenesis and BBB permeability [71], and increased expression of *VEGFA* and its receptor *FLT1* are one of the primary regulators of hypoxia induced angiogenesis and BBB hyperpermeability [42, 50, 74]. *VWF* also promotes BBB hyperpermeability in response to hypoxic conditions [51], and increased expression of *VEGFA*, *FLT1*, and *VWF* was observed in the iron deficient hippocampus. These results, in addition to the enrichment of GO terms and pathways associated with angiogenesis, suggest that iron deficiency resulted in hypoxic conditions leading to increased angiogenesis and BBB permeability. Increased *VWF* expression was also associated with hypomethylation of an intragenic CpG site, suggesting epigenetic regulation of BBB permeability. However, as these results are based solely on bioinformatics analysis, further studies are required to confirm that neonatal iron deficiency resulted in hypoxic conditions, increased angiogenesis, and BBB permeability in the hippocampus.

The enrichment of both up- and down-regulated genes promoting neurodevelopment suggests neonatal iron deficiency leads to altered hippocampal neurodevelopment. This hypothesis is further supported by a recent study reporting decreased white and increased grey matter hippocampal volume in an additional age matched cohort of iron deficient piglets [75]. No difference in total hippocampal volume was observed between groups. Furthermore, increased radial diffusivity and mean diffusivity were increased in the hippocampus of the iron deficient pigs, indicating reduced myelination and less structural organization in the iron deficient hippocampus [75]. Therefore the reported simultaneous up- and down-regulation of genes promoting neurodevelopment, including genes encoding neural and axon guidance cues and receptors, as well as those promoting neurite and axon outgrowth, provides further insight into the mechanisms behind the previously reported alterations in hippocampal neurodevelopment. In addition, the majority of identified DEGs associated with DM sites are involved in neurodevelopment, highlighting the importance of epigenetic regulation in environmentally induced diseases affecting neurodevelopment.

A number of DEGs detected in this study have also been associated with altered neuronal function, including reduced *HTR2A* and increased *HTR2C* expression in individuals suffering from depression and Huntington’s disease [76, 77]. In addition to the decreased *HTR2A* and increased *HTR2C* expression was observed in the iron deficient hippocampus, a high number of DM CpG sites were associated with *HTR2C*, suggesting this gene is epigenetically regulated in response to early life iron deficiency. Additional DEGs down-regulated in the iron deficient hippocampus have been previously implicated in learning and memory functions, including *PAK3*, whose signaling is critical for neuronal connections that underlie cognitive functions [78]. Deficiencies in synaptic plasticity, learning, and memory have also been reported in *Pak3* knock-out mice [79]. In addition, expression of a truncated version of *PRSS12*, another down-regulated DEG, results in symptoms of mental retardation in humans, while *PRSS12* knock outs lead to impaired long-term memory formation in Drosophila and mice [9, 80]. Finally, the synaptic KARs subunit *NETO1* has been implicated in reduced learning and memory in *Neto1* knock out mice [81]. The down-regulation of all three of these genes in the hippocampus of the iron deficient group supports their role in hippocampal-based learning and memory, and suggests the observed reduction in cognitive development is partly due to altered neuronal plasticity and function in the hippocampus.

Although the above mentioned results provide insights into the mechanisms through which neonatal iron deficiency results in reduced cognitive development, the number of identified DEGs associated with DM sites is relatively low (9 DEGs associated with 12 DM sites). The low overlap between the methylation and expression profiles may be due to the limited power of the study caused by the relatively low samples size. Indeed, the low sample size is a limitation of the study, and future studies utilizing additional individuals are required to 1) confirm the results presented here, and 2) further investigate the link between neonatal iron deficiency, hippocampal DNA methylation, and expression. However, it is important to note that while increasing the sample size would likely increase the number of identified DEGs associated with DM sites, another important reason for the relatively low overlap is the lack of functional annotation in the pig reference genome. This lack of knowledge in terms of the location of transcription factor binding sites, promoters, and enhancers makes identifying methylation regulating changes in gene expression difficult. Because of the lack of functional annotation, this study considered a DM site associated with a DEG if it was located within or 10 kb upstream of the gene of interest. While sites located in these regions are likely involved in gene regulation, there are potentially a significant number of DM sites outside these regions that are also involved in regulating gene expression, but are missed due the lack of functional annotation.

Higher similarities in CpG than non-CpG methylation were observed across samples. Consistent with previous results [17], the vast majority of non-CpG sites were unmethylated in the porcine neonatal hippocampus. This, in addition to the higher number of DEGs associated with DM CpG compared to non-CpG sites provides further evidence for CpG methylation as the primary form of regulation in the neonatal brain. The highly conserved accumulation of non-CpG methylation to become the dominate form of gene regulation has been observed during the development of mammalian brain from fetal to adulthood coinciding with synaptogenesis [17]. The higher proportion of unmethylated non-CpG sites in the iron deficient group could indicate a delay in the normal accumulation of non-CpG methylation in response to neonatal iron deficiency. However, increased numbers of methylated (>1 %) non-CpG sites were also detected in the iron deficient group, suggesting an increase in non-CpG methylation accumulation. While it is unclear how neonatal iron deficiency affects the accumulation of non-CpG methylation, samples clustered by group when comparing methylation levels at DM non-CpG sites. This suggests neonatal iron deficiency plays a significant role in shaping non-CpG methylation, which can have a lasting impact on gene regulation and function later in life. In addition, it is interesting that DM CpG sites were found most frequently within gene bodies, while the highest proportion of DM non-CpG sites were located within CpG islands and shores. These results suggest the genomic regions in which neuronal CpG and non-CpG methylation is prone to alteration in response to early life environment varies, and indicates distinct differences in the distribution, regulation, and function of CpG and non-CpG methylation in the developing neonatal hippocampus.

It is important to note that while samples tended to cluster by group when evaluating expression and methylation patterns at DEGs, DM CpG, and DM non-CpG sites, the resulting heat maps still suggest a high level of variability within groups. This is unlikely to be due to phenotypic or sex differences between individuals within groups, as only female individuals displaying cognitive abilities consistent with their group mates were utilized. Female individuals were selected in order to eliminate sex related variability in methylation and expression within groups. However, because of the exclusion of male individuals, further studies incorporating male subjects are required to determine if the reported mechanisms are sex specific, especially given the known effects sex hormones have on hippocampal function and development. The observed reduced cognition phenotype is proposed to be due to altered neurodevelopment and function resulting from a combination of hypoxia-induced angiogenesis and increased BBB permeability, in addition to altered DNA methylation resulting in differential gene expression. Therefore, the degree to which each of these factors contributes to the reduced cognition phenotype may vary between individuals, suggesting different pathways leading to the same phenotype. This could also explain why many of the observed log2 fold changes in expression were relatively low. The variability within groups could also be due to differences in the ratios of grey matter, white matter, and cell types in the hippocampus samples sequenced, as methylation and expression patterns are known to vary across cell types. However, the observation of the reduced cognition phenotype in addition to the clustering of samples by group when assessing differential expression and methylation patterns suggests a consistent effect of neonatal iron deficiency on cognitive development.

## Conclusions

Neonatal iron deficiency resulted in the differential expression of genes involved in response to hypoxia, angiogenesis, and neurodevelopment and function. These results suggest that early life iron deficiency resulted in hypoxia-induced angiogenesis and increased BBB permeability in the hippocampus, the result of an attempt to restore optimal hippocampal iron levels. This most likely led to altered neurodevelopment and function, resulting in the observed reduction in hippocampal-based learning and memory. In addition, methylation analysis revealed genome wide and site-specific differential CpG and non-CpG methylation in response to neonatal iron deficiency. The majority of DEGs associated with DM sites were involved in neurodevelopment and neuronal function, suggesting neonatal iron deficiency results in altered epigenetic regulation of hippocampal neurodevelopment and function, leading to reduced hippocampal-based spatial learning and memory. These results provide insights into the biological mechanisms through which neonatal iron deficiency results in more permanent cognitive deficits in humans.

## Additional file

Additional file 1: Supplemental tables and figures. (DOCX 36770 kb)

## Abbreviations

BBB: Blood brain barrier
CpG sites: Cytosine bases located 5’ adjacent to a guanine
DEGs: Differentially expressed genes
DM: Differentially methylated
gDNA: Genomic DNA
HIFs: Hypoxia-inducible factors
KARs: Kainate receptors
Non-CpG sites: Cytosines outside of CpG contexts
RRBS: Reduced representation bisulfite sequencing
TSS: Transcription start site

## References

[1] Rao R, Georgieff MK (2007). Iron in fetal and neonatal nutrition. Semin Fetal Neonatal Med.

[2] McLean E, Cogswell M, Egli I, Wojdyla D, de Benoist B (2009). Worldwide prevalence of anaemia, WHO vitamin and mineral nutrition information system, 1993-2005. Public Health Nutr.

[3] Lozoff B, Beard J, Connor J, Barbara F, Georgieff M, Schallert T (2006). Long-lasting neural and behavioral effects of iron deficiency in infancy. Nutr Rev.

[4] Georgieff MK (2011). Long-term brain and behavioral consequences of early iron deficiency. Nutr Rev.

[5] Courchesne E, Chisum HJ, Townsend J, Cowles A, Covington J, Egaas B (2000). Normal brain development and aging: quantitative analysis at in vivo MR imaging in healthy volunteers. Radiology.

[6] Gogtay N, Nugent TF, Herman DH, Ordonez A, Greenstein D, Hayashi KM (2006). Dynamic mapping of normal human hippocampal development. Hippocampus.

[7] Vanguilder HD, Bixler GV, Sonntag WE, Freeman WM (2012). Hippocampal expression of myelin-associated inhibitors is induced with age-related cognitive decline and correlates with deficits of spatial learning and memory. J Neurochem.

[8] Bogoch Y, Biala YN, Linial M, Weinstock M (2007). Anxiety induced by prenatal stress is associated with suppression of hippocampal genes involved in synaptic function. J Neurochem.

[9] Mitsui S, Osako Y, Yokoi F, Dang MT, Yuri K, Li Y (2009). A mental retardation gene, motopsin/neurotrypsin/prss12, modulates hippocampal function and social interaction. Eur J Neurosci.

[10] McEwen BS (1999). Stress and hippocampal plasticity. Annu Rev Neurosci.

[11] Goldberg AD, Allis CD, Bernstein E (2007). Epigenetics: a landscape takes shape. Cell.

[12] Ziller MJ, Müller F, Liao J, Zhang Y, Gu H, Bock C, et al. Genomic distribution and inter-sample variation of non-CpG methylation across human cell types. Schübeler D, editor. PLoS Genet. 2011;7:e1002389. 10.1371/journal.pgen.1002389 PMC3234221 22174693

[13] Lister R, Pelizzola M, Dowen RH, Hawkins RD, Hon G, Tonti-Filippini J (2009). Human DNA methylomes at base resolution show widespread epigenomic differences. Nature.

[14] Shirane K, Toh H, Kobayashi H, Miura F, Chiba H, Ito T (2013). Mouse oocyte methylomes at base resolution reveal genome-wide accumulation of non-CpG methylation and role of DNA methyltransferases. PLoS Genet.

[15] Guo JU, Su Y, Shin JH, Shin J, Li H, Xie B (2014). Distribution, recognition and regulation of non-CpG methylation in the adult mammalian brain. Nat Neurosci.

[16] Laine VN, Gossmann TI, Schachtschneider KM, Garroway CJ, Madsen O, Verhoeven KJ (2015). Evolutionary signals of selection on cognition from the great tit genome and methylome. Nat Commun.

[17] Lister R, Mukamel EA, Nery JR, Urich M, Puddifoot CA, Johnson ND (2013). Global epigenomic reconfiguration during mammalian brain development. Science.

[18] Rytych JJL, Elmore MRPM, Burton MMD, Conrad MMS, Donovan SSM, Dilger RNR (2012). Early life iron deficiency impairs spatial cognition in neonatal piglets. J Nutr.

[19] Conrad MS, Johnson RW (2015). The domestic piglet: an important model for investigating the neurodevelopmental consequences of early life insults. Annu Rev Anim Biosci.

[20] Elmore MRP, Dilger RN, Johnson RW (2012). Place and direction learning in a spatial T-maze task by neonatal piglets. Anim Cogn.

[21] Subcommittee on Swine Nutrition, Committee on Animal Nutrition, Board on Agriculture, National Research Council (1998). Nutrient Requirements of Swine. 10th Revis.

[22] Schachtschneider KM, Madsen O, Park C, Rund LA, Groenen MA, Schook LB (2015). Adult porcine genome-wide DNA methylation patterns support pigs as a biomedical model. BMC Genomics.

[23] Choi M, Lee J, Le MT, Nguyen DT, Park S, Soundrarajan N (2015). Genome-wide analysis of DNA methylation in pigs using reduced representation bisulfite sequencing. DNA Res.

[24] Babraham Bioinformatics. http://www.bioinformatics.babraham.ac.uk/projects/trim_galore/. Accessed 5 Nov 2013.

[25] Groenen MAM, Archibald AL, Uenishi H, Tuggle CK, Takeuchi Y, Rothschild MF (2012). Analyses of pig genomes provide insight into porcine demography and evolution. Nature.

[26] Guo W, Fiziev P, Yan W, Cokus S, Sun X, Zhang MQ (2013). BS-Seeker2: a versatile aligning pipeline for bisulfite sequencing data. BMC Genomics.

[27] Langmead B, Salzberg SL (2012). Fast gapped-read alignment with Bowtie 2. Nat Methods.

[28] Akalin A, Kormaksson M, Li S, Garrett-Bakelman FE, Figueroa ME, Melnick A (2012). methylKit: a comprehensive R package for the analysis of genome-wide DNA methylation profiles. Genome Biol.

[29] DePristo MA, Banks E, Poplin R, Garimella KV, Maguire JR, Hartl C (2011). A framework for variation discovery and genotyping using next-generation DNA sequencing data. Nat Genet.

[30] Kim D, Pertea G, Trapnell C, Pimentel H, Kelley R, Salzberg SL (2013). TopHat2: accurate alignment of transcriptomes in the presence of insertions, deletions and gene fusions. Genome Biol.

[31] Trapnell C, Williams BA, Pertea G, Mortazavi A, Kwan G, van Baren MJ (2010). Transcript assembly and quantification by RNA-Seq reveals unannotated transcripts and isoform switching during cell differentiation. Nat Biotechnol.

[32] Schook LB, Collares TV, Hu W, Liang Y, Rodrigues FM, Rund LA (2015). A genetic porcine model of cancer. PLoS One.

[33] Huang DW, Sherman BT, Lempicki RA (2009). Systematic and integrative analysis of large gene lists using DAVID bioinformatics resources. Nat Protoc.

[34] Ogata H, Goto S, Sato K, Fujibuchi W, Bono H, Kanehisa M (1999). KEGG: Kyoto encyclopedia of genes and genomes. Nucleic Acids Res.

[35] Mi H, Poudel S, Muruganujan A, Casagrande JT, Thomas PD (2016). PANTHER version 10: expanded protein families and functions, and analysis tools. Nucleic Acids Res.

[36] Joshi-Tope G, Gillespie M, Vastrik I, D'Eustachio P, Schmidt E, de Bono B (2005). Reactome: a knowledgebase of biological pathways. Nucleic Acids Res.

[37] R Core Team. R: A Language and Environment for Statistical Computing. R Found Stat Comput. 2011. http://www.R-project.org/. Accessed 15 Jan 2015.

[38] Rolfs A, Kvietikova I, Gassmann M, Wenger RH (1997). Oxygen-regulated transferrin expression is mediated by hypoxia-inducible factor-1. J Biol Chem.

[39] Minchenko OH, Kharkova AP, Kubaichuk KI, Minchenko DO, Hlushchak NA, Kovalevska OV (2014). Effect of hypoxia on the expression of CCN2, PLAU, PLAUR, SLURP1, PLAT and ITGB1 genes in ERN1 knockdown U87 glioma cells. Ukr Biochem J.

[40] Zhu Y, Sun Y, Xie L, Jin K, Sheibani N, Greenberg DA (2003). Hypoxic induction of endoglin via mitogen-activated protein kinases in mouse brain microvascular endothelial cells. Stroke.

[41] Li H, Gu B, Zhang Y, Lewis DF, Wang Y (2005). Hypoxia-induced increase in soluble Flt-1 production correlates with enhanced oxidative stress in trophoblast cells from the human placenta. Placenta.

[42] Liu Y, Cox SR, Morita T, Kourembanas S (1995). Hypoxia regulates vascular endothelial growth factor gene expression in endothelial cells : identification of a 5’ enhancer. Circ Res.

[43] van Meeteren LA, Ruurs P, Stortelers C, Bouwman P, van Rooijen MA, Pradère JP (2006). Autotaxin, a secreted lysophospholipase D, is essential for blood vessel formation during development. Mol Cell Biol.

[44] Nichol D, Stuhlmann H (2012). EGFL7: a unique angiogenic signaling factor in vascular development and disease. Blood.

[45] Kamisasanuki T, Tokushige S, Terasaki H, Khai NC, Wang Y, Sakamoto T (2011). Targeting CD9 produces stimulus-independent antiangiogenic effects predominantly in activated endothelial cells during angiogenesis: a novel antiangiogenic therapy. Biochem Biophys Res Commun.

[46] Li DY (1999). Defective angiogenesis in mice lacking endoglin. Science.

[47] Hiratsuka S, Minowa O, Kuno J, Noda T, Shibuya M (1998). Flt-1 lacking the tyrosine kinase domain is sufficient for normal development and angiogenesis in mice. Proc Natl Acad Sci.

[48] Ferrara N (2000). VEGF: an update on biological and therapeutic aspects. Curr Opin Biotechnol.

[49] Oehler MK, Hague S, Rees MCP, Bicknell R (2002). Adrenomedullin promotes formation of xenografted endometrial tumors by stimulation of autocrine growth and angiogenesis. Oncogene.

[50] Fischer S, Clauss M, Wiesnet M, Renz D, Schaper W, Karliczek GF (1999). Hypoxia induces permeability in brain microvessel endothelial cells via VEGF and NO. Am J Physiol.

[51] Suidan GL, Brill A, De Meyer SF, Voorhees JR, Cifuni SM, Cabral JE (2013). Endothelial Von Willebrand factor promotes blood-brain barrier flexibility and provides protection from hypoxia and seizures in mice. Arterioscler Thromb Vasc Biol.

[52] Johansson S, Povlsen GK, Edvinsson L (2012). Expressional changes in cerebrovascular receptors after experimental transient forebrain ischemia. PLoS One.

[53] Nakamura F, Kalb RG, Strittmatter SM (2000). Molecular basis of semaphorin-mediated axon guidance. J Neurobiol.

[54] Lin JC, Ho W-H, Gurney A, Rosenthal A (2003). The netrin-G1 ligand NGL-1 promotes the outgrowth of thalamocortical axons. Nat Neurosci.

[55] Zhang J, Wang S, Yuan L, Yang Y, Zhang B, Liu Q (2012). Neuron-restrictive silencer factor (NRSF) represses cocaine- and amphetamine-regulated transcript (CART) transcription and antagonizes cAMP-response element-binding protein signaling through a dual NRSE mechanism. J Biol Chem.

[56] Ba-Charvet KTN, Brose K, Marillat V, Kidd T, Goodman CS, Tessier-Lavigne M (1999). Slit2-mediated chemorepulsion and collapse of developing forebrain axons. Neuron.

[57] Yamagishi S, Hampel F, Hata K, Del Toro D, Schwark M, Kvachnina E (2011). FLRT2 and FLRT3 act as repulsive guidance cues for Unc5-positive neurons. EMBO J.

[58] Hata K, Kaibuchi K, Inagaki S, Yamashita T (2009). Unc5B associates with LARG to mediate the action of repulsive guidance molecule. J Cell Biol.

[59] Taniguchi M, Masuda T, Fukaya M, Kataoka H, Mishina M, Yaginuma H (2005). Identification and characterization of a novel member of murine semaphorin family. Genes Cells.

[60] Hartwig C, Veske A, Krejcova S, Rosenberger G, Finckh U (2005). Plexin B3 promotes neurite outgrowth, interacts homophilically, and interacts with Rin. BMC Neurosci.

[61] Seeds NW, Basham ME, Haffke SP (1999). Neuronal migration is retarded in mice lacking the tissue plasminogen activator gene. Proc Natl Acad Sci.

[62] Cavallaro S (2008). Genomic analysis of serotonin receptors in learning and memory. Behav Brain Res.

[63] Boda B, Alberi S, Nikonenko I, Node-Langlois R, Jourdain P, Moosmayer M (2004). The mental retardation protein PAK3 contributes to synapse formation and plasticity in hippocampus. J Neurosci.

[64] Mitsui S, Yamaguchi N, Osako Y, Yuri K (2007). Enzymatic properties and localization of motopsin (PRSS12), a protease whose absence causes mental retardation. Brain Res.

[65] Tang M, Pelkey KA, Ng D, Ivakine E, McBain CJ, Salter MW (2011). Neto1 is an auxiliary subunit of native synaptic kainate receptors. J Neurosci.

[66] Fryxell KJ, Moon W-J (2005). CpG mutation rates in the human genome are highly dependent on local GC content. Mol Biol Evol.

[67] Spruijt CG, Vermeulen M (2014). DNA methylation: old dog, new tricks?. Nat Struct Mol Biol.

[68] Janz R, Goda Y, Geppert M, Missler M, Südhof TC (1999). SV2A and SV2B function as redundant Ca2+ regulators in neurotransmitter release. Neuron.

[69] Sakane A, Honda K, Sasaki T (2010). Rab13 regulates neurite outgrowth in PC12 cells through its effector protein, JRAB/MICAL-L2. Mol Cell Biol.

[70] Han J, Day JR, Connor JR, Beard JL (2003). Gene expression of transferrin and transferrin receptor in brains of control vs. Iron-deficient rats. Nutr Neurosci.

[71] Peyssonnaux C, Zinkernagel AS, Schuepbach RA, Rankin E, Vaulont S, Haase VH (2007). Regulation of iron homeostasis by the hypoxia-inducible transcription factors (HIFs). J Clin Invest.

[72] Todorich B, Pasquini JM, Garcia CI, Paez PM, Connor JR (2009). Oligodendrocytes and myelination: the role of iron. Glia.

[73] Salmaso N, Jablonska B, Scafidi J, Vaccarino FM, Gallo V (2014). Neurobiology of premature brain injury. Nat Neurosci.

[74] Yeh WL, Lu DY, Lin CJ, Liou HC, Fu WM. Inhibition of hypoxia-induced increase of blood-brain barrier permeability by YC-1 through the antagonism of HIF-1alpha accumulation and VEGF expression. Mol Pharmacol. 2007;72:440–9. 10.1124/mol.107.036418 17513385

[75] Leyshon BJ, Radlowski EC, Mudd AT, Steelman AJ, Johnson RW (2016). Postnatal iron deficiency alters brain development in piglets. J Nutr.

[76] Pang TYC, Du X, Zajac MS, Howard ML, Hannan AJ (2009). Altered serotonin receptor expression is associated with depression-related behavior in the R6/1 transgenic mouse model of Huntington’s disease. Hum Mol Genet.

[77] Duric V, Banasr M, Stockmeier CA, Simen AA, Newton SS, Overholser JC (2013). Altered expression of synapse and glutamate related genes in post-mortem hippocampus of depressed subjects. Int J Neuropsychopharmacol.

[78] Allen KM, Gleeson JG, Bagrodia S, Partington MW, MacMillan JC, Cerione RA (1998). PAK3 mutation in nonsyndromic X-linked mental retardation. Nat Genet.

[79] Meng J, Meng Y, Hanna A, Janus C, Jia Z (2005). Abnormal long-lasting synaptic plasticity and cognition in mice lacking the mental retardation gene Pak3. J Neurosci.

[80] Didelot G, Molinari F, Tche P, Comas D, Milhiet E, Munnich E, et al. Tequila, a Neurotrypsin Ortholog, Regulates Long-Term Memory Formation in Drosophila. Science. 2006;313:2005–7 10.1126/science.1127215 16902143

[81] Ng D, Pitcher GM, Szilard RK, Sertié A, Kanisek M, Clapcote SJ (2009). Neto1 is a novel CUB-domain NMDA receptor-interacting protein required for synaptic plasticity and learning. PLoS Biol.

