# Supplementary material for: Impact of neonatal iron deficiency on hippocampal DNA methylation and gene transcription in a porcine biomedical model of cognitive development
Source: BMC Genomics. 2016 Nov 3;17:856. doi: 10.1186/s12864-016-3216-y (PMC5094146; doi:10.1186/s12864-016-3216-y)
Supplement: Additional file 1: — Supplemental tables and figures. (DOCX 36770 kb) [file 12864_2016_3216_MOESM1_ESM.docx]

**Table of Contents**

Table S1. RRBS, targeted control, and RNA-seq sequencing depths for all samples

Table S2. RRBS and targeted control library genome coverage and depth

Table S3. List of DEGs up-regulated in the iron deficient group

Table S4. List of DEGs down-regulated in the iron deficient group

Table S5. Enriched GO terms and pathways for DEGs detected between groups

Table S6. Enriched GO terms and pathways for DEGs up-regulated in the iron deficient group

Table S7. Enriched GO terms and pathways for DEGs down-regulated in the iron deficient group

Figure S1. Evidence of iron deficiency and reduced cognition in the iron deficient pigs.

Figure S2. Correlation analysis of CpG methylation levels

Figure S3. Correlation analysis of non-CpG methylation levels

| **Table S1. RRBS, targeted control, and RNA-seq sequencing depths for all samples** | | | | | |
| --- | --- | --- | --- | --- | --- |
| **Pig #** | **Treatment** | **RRBS (1 x 100 bp) Total Reads** | **Targeted control (1 x 100 bp) Total Reads** | **RNA-seq (2 x 100 bp) Total Read Pairs** | **RNA Integrity Number (RIN)** |
| **13** | Control | 60,489,724 | 15,083,236 | 30,384,386 | 9.8 |
| **19** | Control | 58,172,989 | 18,503,830 | 29,789,916 | 9.7 |
| **25** | Control | 58,297,937 | 17,050,799 | 37,146,274 | 9.2 |
| **31** | Control | 61,128,020 | 16,720,509 | 33,922,473 | 9.4 |
| **15** | Severe Iron Deficient | 37,621,615 | 12,603,025 | 34,083,364 | 7.4 |
| **33** | Severe Iron Deficient | 39,283,187 | 13,670,048 | 39,013,189 | 9.6 |
| **27** | Severe Iron Deficient | 50,932,886 | 15,090,630 | 42,831,034 | 9.9 |

| **Table S2. Targeted control and RRBS genome coverage and depth** | | | | | |
| --- | --- | --- | --- | --- | --- |
|  |  | **Targeted Control** | | **RRBS** | |
| **Pig #** | **Treatment** | **% Genome Coverage** | **Depth** | **% Genome Coverage** | **Depth** |
| **13** | Control | 1.07 | 6.93 | 1.37 | 22.25 |
| **19** | Control | 1.23 | 9.16 | 1.25 | 30.02 |
| **25** | Control | 1.37 | 7.73 | 1.35 | 27.32 |
| **31** | Control | 1.74 | 5.93 | 1.50 | 24.42 |
| **15** | Severe Iron Deficient | 0.97 | 6.73 | 1.14 | 17.47 |
| **33** | Severe Iron Deficient | 1.30 | 6.55 | 1.27 | 20.59 |
| **27** | Severe Iron Deficient | 1.27 | 7.15 | 1.27 | 24.78 |

| **Table S3. List of DEGs Up-regulated in the iron deficient group** | | | | |
| --- | --- | --- | --- | --- |
| **Genes** | **Control (FPKM)** | **Deficient (FPKM)** | **log2 fold change** | **q-value** |
| ENSSSCG00000026802 | 0.07 | 1.21 | 4.22 | 0.00965528 |
| CU207250.1 | 0.25 | 2.63 | 3.40 | 0.00965528 |
| NPHS2 | 0.15 | 1.27 | 3.08 | 0.00965528 |
| CHD8 | 10.83 | 87.58 | 3.02 | 0.00965528 |
| NMBR | 0.10 | 0.60 | 2.56 | 0.0472776 |
| TTR | 4.16 | 23.40 | 2.49 | 0.00965528 |
| ENSSSCG00000028560 | 7.15 | 39.82 | 2.48 | 0.00965528 |
| ADM | 0.55 | 2.73 | 2.31 | 0.00965528 |
| ENSSSCG00000026923 | 9.71 | 44.64 | 2.20 | 0.00965528 |
| NTS | 5.33 | 24.24 | 2.18 | 0.00965528 |
| SERPINE1 | 0.60 | 2.55 | 2.08 | 0.0275588 |
| VWF | 2.61 | 9.18 | 1.81 | 0.00965528 |
| ENSSSCG00000010493 | 0.79 | 2.74 | 1.80 | 0.00965528 |
| PGF | 0.99 | 3.45 | 1.79 | 0.00965528 |
| SMAD6 | 0.81 | 2.79 | 1.78 | 0.00965528 |
| S100A3 | 3.10 | 10.57 | 1.77 | 0.0170317 |
| SERPINA1 | 1.89 | 6.38 | 1.75 | 0.00965528 |
| CLEC2B | 1.08 | 3.57 | 1.72 | 0.00965528 |
| AOC3 | 0.56 | 1.83 | 1.70 | 0.00965528 |
| TAGLN | 14.04 | 43.71 | 1.64 | 0.00965528 |
| COL11A2 | 1.83 | 5.62 | 1.62 | 0.00965528 |
| MYL9 | 4.88 | 14.50 | 1.57 | 0.00965528 |
| ACER2 | 2.40 | 7.07 | 1.56 | 0.00965528 |
| ACSM5 | 0.48 | 1.40 | 1.56 | 0.0275588 |
| ACTA2 | 11.45 | 33.43 | 1.55 | 0.00965528 |
| GPR126 | 0.75 | 2.17 | 1.54 | 0.00965528 |
| ENSSSCG00000027544 | 3.28 | 9.28 | 1.50 | 0.00965528 |
| MNDA | 1.31 | 3.63 | 1.47 | 0.00965528 |
| LRRC32 | 0.65 | 1.79 | 1.46 | 0.00965528 |
| TMEM107 | 6.08 | 16.40 | 1.43 | 0.0170317 |
| COL18A1 | 6.06 | 16.18 | 1.42 | 0.00965528 |
| FOXF2 | 0.76 | 2.01 | 1.41 | 0.0430133 |
| ANXA13 | 1.62 | 4.22 | 1.39 | 0.00965528 |
| SMIM15 | 23.37 | 60.90 | 1.38 | 0.0170317 |
| DLL4 | 1.34 | 3.49 | 1.38 | 0.00965528 |
| A4GALT | 2.27 | 5.89 | 1.38 | 0.0224762 |
| MGP | 14.15 | 36.24 | 1.36 | 0.00965528 |
| ALPL | 7.76 | 19.66 | 1.34 | 0.00965528 |
| PPP1R11 | 1.27 | 3.19 | 1.34 | 0.0404951 |
| PAPPA | 0.30 | 0.74 | 1.30 | 0.00965528 |
| TMEM26 | 5.89 | 14.39 | 1.29 | 0.00965528 |
| EDN1 | 1.38 | 3.33 | 1.27 | 0.0224762 |
| CD248 | 1.14 | 2.72 | 1.26 | 0.00965528 |
| ENSSSCG00000020978 | 1.74 | 4.14 | 1.25 | 0.0404951 |
| AADC | 2.13 | 5.06 | 1.25 | 0.0368892 |
| MICALL2 | 2.74 | 6.46 | 1.24 | 0.00965528 |
| VEGFA | 6.33 | 14.83 | 1.23 | 0.00965528 |
| ARRDC2 | 4.11 | 9.52 | 1.21 | 0.00965528 |
| FLT1 | 13.49 | 31.16 | 1.21 | 0.00965528 |
| SH3TC2 | 2.30 | 5.22 | 1.18 | 0.00965528 |
| COL4A1 | 21.28 | 47.80 | 1.17 | 0.00965528 |
| PLAT | 11.95 | 26.66 | 1.16 | 0.00965528 |
| MYH11 | 2.10 | 4.65 | 1.15 | 0.0368892 |
| CYP26B1 | 1.23 | 2.66 | 1.12 | 0.00965528 |
| GATA2 | 1.81 | 3.90 | 1.11 | 0.0170317 |
| ENG | 7.17 | 15.40 | 1.10 | 0.00965528 |
| ENSSSCG00000025146 | 5.59 | 11.98 | 1.10 | 0.00965528 |
| BCAM | 8.41 | 17.95 | 1.09 | 0.00965528 |
| COL4A2 | 15.22 | 32.41 | 1.09 | 0.00965528 |
| HTR2C | 3.19 | 6.74 | 1.08 | 0.00965528 |
| CLDN5 | 39.01 | 80.45 | 1.04 | 0.0324893 |
| TAGLN2 | 8.80 | 18.13 | 1.04 | 0.00965528 |
| EVA1C | 4.04 | 8.17 | 1.02 | 0.0170317 |
| CTGF | 13.10 | 26.37 | 1.01 | 0.00965528 |
| ENSSSCG00000027611 | 1.36 | 2.71 | 1.00 | 0.0324893 |
| CD93 | 5.04 | 10.03 | 0.99 | 0.00965528 |
| MYLK | 4.95 | 9.85 | 0.99 | 0.0404951 |
| CENPE | 1.67 | 3.33 | 0.99 | 0.0224762 |
| SEMA3G | 5.30 | 10.52 | 0.99 | 0.00965528 |
| PLEKHG3 | 9.82 | 19.43 | 0.98 | 0.00965528 |
| ACKR3 | 6.01 | 11.88 | 0.98 | 0.00965528 |
| PCDH12 | 1.41 | 2.79 | 0.98 | 0.0368892 |
| GGT5 | 2.16 | 4.25 | 0.98 | 0.0472776 |
| TPM2 | 9.02 | 17.71 | 0.97 | 0.0224762 |
| ENSSSCG00000020777 | 3.12 | 6.03 | 0.95 | 0.0324893 |
| ENSSSCG00000003514 | 2.37 | 4.57 | 0.95 | 0.00965528 |
| TF | 106.01 | 203.90 | 0.94 | 0.00965528 |
| ENSSSCG00000014106 | 19.16 | 36.63 | 0.94 | 0.0275588 |
| CD82 | 10.38 | 19.79 | 0.93 | 0.00965528 |
| SLA1 | 47.28 | 90.05 | 0.93 | 0.00965528 |
| TMC7 | 4.06 | 7.61 | 0.90 | 0.0275588 |
| CLEC14A | 2.82 | 5.27 | 0.90 | 0.0368892 |
| EHD2 | 4.01 | 7.40 | 0.89 | 0.0430133 |
| MAL | 34.96 | 64.58 | 0.89 | 0.0368892 |
| PXDC1 | 9.35 | 17.26 | 0.88 | 0.00965528 |
| MAG | 123.33 | 227.18 | 0.88 | 0.00965528 |
| SMTN | 4.61 | 8.45 | 0.87 | 0.0224762 |
| DCHS1 | 4.04 | 7.40 | 0.87 | 0.0430133 |
| OPALIN | 94.09 | 171.75 | 0.87 | 0.0170317 |
| SLC52A3 | 4.98 | 9.03 | 0.86 | 0.0324893 |
| DUSP1 | 9.39 | 16.97 | 0.85 | 0.0170317 |
| PLXNB3 | 9.20 | 16.57 | 0.85 | 0.00965528 |
| CD9 | 52.69 | 94.73 | 0.85 | 0.00965528 |
| EGFL7 | 10.68 | 19.19 | 0.85 | 0.0275588 |
| VWA1 | 13.07 | 23.43 | 0.84 | 0.00965528 |
| ENSSSCG00000020869 | 46.48 | 83.04 | 0.84 | 0.0275588 |
| ENPP2 | 65.31 | 116.58 | 0.84 | 0.0368892 |
| PECAM1 | 10.10 | 17.87 | 0.82 | 0.0404951 |
| LENG8 | 29.97 | 52.81 | 0.82 | 0.0275588 |
| ENSSSCG00000021844 | 219.13 | 385.34 | 0.81 | 0.0224762 |
| SGK2 | 11.43 | 19.95 | 0.80 | 0.0368892 |
| TMEM144 | 24.43 | 42.64 | 0.80 | 0.00965528 |
| ENSSSCG00000000263 | 8.13 | 14.07 | 0.79 | 0.0170317 |
| MYRF | 14.68 | 25.25 | 0.78 | 0.0224762 |
| ENSSSCG00000026635 | 43.77 | 75.16 | 0.78 | 0.0224762 |
| PLCL1 | 6.15 | 10.52 | 0.77 | 0.0404951 |
| ENSSSCG00000021418 | 23.28 | 39.77 | 0.77 | 0.00965528 |
| TTYH2 | 31.94 | 54.42 | 0.77 | 0.0224762 |
| FBXO32 | 12.86 | 21.83 | 0.76 | 0.0170317 |
| RAMP2 | 45.62 | 77.38 | 0.76 | 0.0170317 |
| ELOVL1 | 6.33 | 10.73 | 0.76 | 0.0430133 |
| ERMN | 103.65 | 174.34 | 0.75 | 0.0430133 |
| TXNIP | 20.46 | 34.29 | 0.75 | 0.0430133 |
| PLA2R1 | 9.96 | 16.56 | 0.73 | 0.0224762 |
| PLLP | 85.62 | 142.38 | 0.73 | 0.0275588 |
| GPR116 | 7.48 | 12.40 | 0.73 | 0.0368892 |
| SEPT4_ | 94.78 | 156.10 | 0.72 | 0.0430133 |
| UNC5B | 7.68 | 12.56 | 0.71 | 0.0430133 |
| TTYH1 | 76.08 | 135.31 | 0.70 | 0.0324893 |
| ENSSSCG00000012039 | 35.73 | 57.20 | 0.68 | 0.0170317 |
| KIF1C | 14.78 | 23.48 | 0.67 | 0.0430133 |

| **Table S4. List of DEGs down-regulated in the iron defecient group** | | | | |
| --- | --- | --- | --- | --- |
| **Genes** | **Control (FPKM)** | **Deficient (FPKM)** | **log2 fold change** | **q-value** |
| U6 | 1.63 | 0.10 | -4.03 | 0.00965528 |
| ENSSSCG00000006881 | 13.36 | 2.63 | -2.34 | 0.0170317 |
| TMPCH242-74M17.6 | 51.38 | 10.35 | -2.31 | 0.00965528 |
| GLRA3 | 4.16 | 0.96 | -2.11 | 0.00965528 |
| CARTPT | 136.00 | 32.29 | -2.07 | 0.00965528 |
| OLFM4 | 2.14 | 0.58 | -1.89 | 0.00965528 |
| C7 | 2.65 | 0.72 | -1.87 | 0.00965528 |
| GABRE | 2.85 | 0.85 | -1.75 | 0.0170317 |
| LOX | 6.33 | 1.88 | -1.75 | 0.00965528 |
| ENSSSCG00000016159 | 0.74 | 0.23 | -1.67 | 0.0430133 |
| ENSSSCG00000023256 | 14.77 | 4.87 | -1.60 | 0.0275588 |
| ENSSSCG00000005951 | 2.15 | 0.75 | -1.52 | 0.00965528 |
| ENSSSCG00000015458 | 300.84 | 106.85 | -1.49 | 0.00965528 |
| NPY2R | 5.88 | 2.25 | -1.39 | 0.00965528 |
| SEMA3E | 17.74 | 6.94 | -1.35 | 0.00965528 |
| AQP3 | 18.95 | 7.51 | -1.34 | 0.00965528 |
| HS6ST2 | 21.37 | 8.64 | -1.31 | 0.0368892 |
| HTR2A | 2.24 | 0.93 | -1.28 | 0.00965528 |
| PLCXD3 | 23.64 | 10.03 | -1.24 | 0.00965528 |
| ENSSSCG00000027841 | 73.91 | 33.05 | -1.16 | 0.00965528 |
| CD24 | 106.98 | 47.97 | -1.16 | 0.00965528 |
| COCH | 16.15 | 7.33 | -1.14 | 0.00965528 |
| NR2F2 | 23.48 | 10.83 | -1.12 | 0.00965528 |
| STMN1 | 978.55 | 455.14 | -1.10 | 0.00965528 |
| FILIP1 | 14.11 | 6.62 | -1.09 | 0.00965528 |
| NTNG1 | 15.24 | 7.16 | -1.09 | 0.00965528 |
| DUSP5 | 8.80 | 4.17 | -1.08 | 0.00965528 |
| ASB18 | 4.60 | 2.21 | -1.05 | 0.0404951 |
| ENSSSCG00000000436 | 4.67 | 2.25 | -1.05 | 0.00965528 |
| NETO1 | 40.98 | 19.88 | -1.04 | 0.00965528 |
| KCNQ5 | 13.58 | 6.59 | -1.04 | 0.00965528 |
| TRHDE | 18.35 | 8.93 | -1.04 | 0.00965528 |
| AK5 | 220.10 | 108.31 | -1.02 | 0.00965528 |
| TNFAIP8 | 4.96 | 2.45 | -1.02 | 0.0324893 |
| FIBCD1 | 58.82 | 29.63 | -0.99 | 0.0224762 |
| SV2B | 61.27 | 31.34 | -0.97 | 0.00965528 |
| ENSSSCG00000027183 | 12.42 | 6.37 | -0.96 | 0.0275588 |
| ENSSSCG00000024107 | 103.23 | 53.26 | -0.95 | 0.00965528 |
| CDH9 | 14.24 | 7.44 | -0.94 | 0.00965528 |
| PRSS12 | 19.31 | 10.11 | -0.93 | 0.00965528 |
| F5 | 35.44 | 18.75 | -0.92 | 0.00965528 |
| ENSSSCG00000022272 | 9.84 | 5.21 | -0.92 | 0.00965528 |
| KIT | 53.55 | 28.64 | -0.90 | 0.00965528 |
| FAM105A | 33.17 | 17.79 | -0.90 | 0.0170317 |
| NOR1 | 11.07 | 5.96 | -0.89 | 0.00965528 |
| AKAP12 | 12.99 | 6.99 | -0.89 | 0.0170317 |
| DCHS2 | 2.29 | 1.24 | -0.89 | 0.0224762 |
| SERTM1 | 33.95 | 18.35 | -0.89 | 0.0275588 |
| SLIT2 | 22.32 | 12.15 | -0.88 | 0.00965528 |
| ENSSSCG00000008970 | 18.46 | 10.07 | -0.87 | 0.00965528 |
| SLC7A14 | 26.56 | 14.60 | -0.86 | 0.00965528 |
| EDNRB | 5.27 | 2.91 | -0.86 | 0.0472776 |
| AEBP1 | 30.09 | 16.63 | -0.86 | 0.00965528 |
| VWA5A | 21.62 | 12.01 | -0.85 | 0.00965528 |
| UNC5D | 7.70 | 4.29 | -0.85 | 0.0404951 |
| ENTPD4 | 15.39 | 8.62 | -0.84 | 0.00965528 |
| EPDR1 | 49.66 | 28.45 | -0.80 | 0.00965528 |
| GDA | 89.38 | 51.29 | -0.80 | 0.0324893 |
| PCSK2 | 50.16 | 28.83 | -0.80 | 0.00965528 |
| PAK3 | 73.77 | 42.56 | -0.79 | 0.0324893 |
| TTC39B | 5.28 | 3.05 | -0.79 | 0.0275588 |
| FRRS1L | 32.25 | 18.67 | -0.79 | 0.00965528 |
| ENSSSCG00000022821 | 78.68 | 45.93 | -0.78 | 0.0170317 |
| C9orf72 | 15.85 | 9.30 | -0.77 | 0.0224762 |
| CADPS2 | 20.43 | 12.15 | -0.75 | 0.0324893 |
| RAB9B | 15.86 | 9.52 | -0.74 | 0.0275588 |
| PPP4R4 | 14.66 | 8.88 | -0.72 | 0.0430133 |
| SH3GL2 | 75.08 | 45.67 | -0.72 | 0.0324893 |
| B3GALNT1 | 43.01 | 26.24 | -0.71 | 0.0275588 |
| ENSSSCG00000016073 | 23.07 | 14.23 | -0.70 | 0.0224762 |
| CLVS2 | 19.09 | 11.80 | -0.69 | 0.0430133 |

| **Table S5. Enriched GO terms and pathways for DEGs detected between groups** | | | | | |
| --- | --- | --- | --- | --- | --- |
| **Category** | **Term** | **Count** | **%** | **Fold Enrichment** | **Benjamini** |
| GOTERM_BP_FAT | GO:0001525~angiogenesis | 10 | 6.67 | 7.95 | 1.31E-03 |
| GOTERM_BP_FAT | GO:0001568~blood vessel development | 13 | 8.67 | 6.24 | 1.25E-03 |
| GOTERM_BP_FAT | GO:0001569~patterning of blood vessels | 4 | 2.67 | 22.41 | 0.039590388 |
| GOTERM_BP_FAT | GO:0001666~response to hypoxia | 9 | 6.00 | 7.90 | 0.004179731 |
| GOTERM_BP_FAT | GO:0001763~morphogenesis of a branching structure | 7 | 4.67 | 11.13 | 4.94E-03 |
| GOTERM_BP_FAT | GO:0001944~vasculature development | 13 | 8.67 | 6.09 | 8.09E-04 |
| GOTERM_BP_FAT | GO:0003013~circulatory system process | 10 | 6.67 | 6.32 | 4.72E-03 |
| GOTERM_BP_FAT | GO:0006939~smooth muscle contraction | 5 | 3.33 | 17.82 | 0.01404937 |
| GOTERM_BP_FAT | GO:0007155~cell adhesion | 18 | 12.00 | 3.02 | 0.007642841 |
| GOTERM_BP_FAT | GO:0008015~blood circulation | 10 | 6.67 | 6.32 | 4.72E-03 |
| GOTERM_BP_FAT | GO:0009611~response to wounding | 14 | 9.33 | 3.11 | 0.034256594 |
| GOTERM_BP_FAT | GO:0022610~biological adhesion | 18 | 12.00 | 3.02 | 0.007181497 |
| GOTERM_BP_FAT | GO:0030198~extracellular matrix organization | 7 | 4.67 | 7.92 | 0.017348243 |
| GOTERM_BP_FAT | GO:0030334~regulation of cell migration | 8 | 5.33 | 5.57 | 0.033799216 |
| GOTERM_BP_FAT | GO:0040012~regulation of locomotion | 10 | 6.67 | 6.13 | 0.005304384 |
| GOTERM_BP_FAT | GO:0048514~blood vessel morphogenesis | 12 | 8.00 | 6.69 | 6.59E-04 |
| GOTERM_BP_FAT | GO:0048754~branching morphogenesis of a tube | 6 | 4.00 | 10.86 | 0.017352598 |
| GOTERM_BP_FAT | GO:0070482~response to oxygen levels | 9 | 6.00 | 7.51 | 5.04E-03 |
| GOTERM_CC_FAT | GO:0005576~extracellular region | 41 | 27.33 | 2.10 | 0.000152449 |
| GOTERM_CC_FAT | GO:0005578~proteinaceous extracellular matrix | 14 | 9.33 | 4.51 | 0.00035723 |
| GOTERM_CC_FAT | GO:0005604~basement membrane | 6 | 4.00 | 7.93 | 0.017571813 |
| GOTERM_CC_FAT | GO:0005615~extracellular space | 23 | 15.33 | 3.46 | 4.64836E-05 |
| GOTERM_CC_FAT | GO:0030141~secretory granule | 11 | 7.33 | 6.30 | 0.000315669 |
| GOTERM_CC_FAT | GO:0031012~extracellular matrix | 15 | 10.00 | 4.48 | 0.000245916 |
| GOTERM_CC_FAT | GO:0044420~extracellular matrix part | 8 | 5.33 | 7.05 | 0.00330261 |
| GOTERM_CC_FAT | GO:0044421~extracellular region part | 32 | 21.33 | 3.44 | 2.19365E-07 |
| GOTERM_MF_FAT | GO:0005509~calcium ion binding | 22 | 14.67 | 2.88 | 0.004798578 |
| GOTERM_MF_FAT | GO:0030246~carbohydrate binding | 12 | 8.00 | 4.08 | 0.023332916 |
| KEGG_PATHWAY | hsa04510:Focal adhesion | 9 | 6.00 | 3.79 | 0.037168217 |
| REACTOME_PATHWAY | REACT_12529:Signaling by VEGF | 3 | 2.00 | 26.48 | 0.044559813 |
| REACTOME_PATHWAY | REACT_604:Hemostasis | 9 | 6.00 | 3.72 | 0.030929465 |
| GOTERM_BP_FAT | GO:0031644~regulation of neurological system process | 9 | 6.00 | 6.92 | 0.005429394 |
| GOTERM_BP_FAT | GO:0035295~tube development | 9 | 6.00 | 4.81 | 0.034214284 |
| GOTERM_BP_FAT | GO:0044057~regulation of system process | 12 | 8.00 | 4.57 | 0.00650751 |
| GOTERM_BP_FAT | GO:0051969~regulation of transmission of nerve impulse | 8 | 5.33 | 6.40 | 0.018310448 |
| GOTERM_CC_FAT | GO:0005886~plasma membrane | 56 | 37.33 | 1.53 | 0.00532554 |
| GOTERM_CC_FAT | GO:0031091~platelet alpha granule | 5 | 3.33 | 9.20 | 0.029153008 |
| GOTERM_CC_FAT | GO:0031226~intrinsic to plasma membrane | 23 | 15.33 | 1.95 | 0.035278181 |
| GOTERM_CC_FAT | GO:0031410~cytoplasmic vesicle | 16 | 10.67 | 2.57 | 0.021697525 |
| GOTERM_CC_FAT | GO:0031982~vesicle | 16 | 10.67 | 2.46 | 0.029911709 |
| KEGG_PATHWAY | hsa04360:Axon guidance | 8 | 5.33 | 5.26 | 0.035966005 |
| KEGG_PATHWAY | hsa04610:Complement and coagulation cascades | 6 | 4.00 | 7.37 | 0.031106195 |

| **Table S6. Enriched GO terms and pathways for DEGs up-regulated in the iron deficient group** | | | | | | |
| --- | --- | --- | --- | --- | --- | --- |
| **Category** | **Term** | **Count** | **%** | **Fold Enrichment** | | **Benjamini** |
| GOTERM_BP_FAT | GO:0001525~angiogenesis | 9 | 9.18 | 10.82 | 0.0004923 | |
| GOTERM_BP_FAT | GO:0001568~blood vessel development | 10 | 10.20 | 7.27 | 0.001280929 | |
| GOTERM_BP_FAT | GO:0001569~patterning of blood vessels | 4 | 4.08 | 33.90 | 0.01922411 | |
| GOTERM_BP_FAT | GO:0001666~response to hypoxia | 9 | 9.18 | 11.96 | 0.000695056 | |
| GOTERM_BP_FAT | GO:0001763~morphogenesis of a branching structure | 7 | 7.14 | 16.84 | 0.000623851 | |
| GOTERM_BP_FAT | GO:0001944~vasculature development | 10 | 10.20 | 7.09 | 0.001334055 | |
| GOTERM_BP_FAT | GO:0003013~circulatory system process | 7 | 7.14 | 6.70 | 0.03585886 | |
| GOTERM_MF_FAT | GO:0005509~calcium ion binding | 16 | 16.33 | 3.01 | 0.012978332 | |
| GOTERM_MF_FAT | GO:0005529~sugar binding | 8 | 8.16 | 7.10 | 0.013188289 | |
| GOTERM_CC_FAT | GO:0005576~extracellular region | 25 | 25.51 | 1.89 | 0.030897085 | |
| GOTERM_CC_FAT | GO:0005578~proteinaceous extracellular matrix | 11 | 11.22 | 5.23 | 0.003052905 | |
| GOTERM_CC_FAT | GO:0005604~basement membrane | 6 | 6.12 | 11.71 | 0.005603673 | |
| GOTERM_CC_FAT | GO:0005615~extracellular space | 14 | 14.29 | 3.11 | 0.013217737 | |
| GOTERM_BP_FAT | GO:0006939~smooth muscle contraction | 4 | 4.08 | 21.58 | 0.039179289 | |
| GOTERM_BP_FAT | GO:0007155~cell adhesion | 13 | 13.27 | 3.31 | 0.031575389 | |
| GOTERM_BP_FAT | GO:0008015~blood circulation | 7 | 7.14 | 6.70 | 0.03585886 | |
| GOTERM_BP_FAT | GO:0009611~response to wounding | 11 | 11.22 | 3.69 | 0.038566495 | |
| GOTERM_BP_FAT | GO:0022610~biological adhesion | 13 | 13.27 | 3.30 | 0.029727375 | |
| GOTERM_CC_FAT | GO:0030141~secretory granule | 7 | 7.14 | 5.92 | 0.023552333 | |
| GOTERM_BP_FAT | GO:0030198~extracellular matrix organization | 6 | 6.12 | 10.27 | 0.023634538 | |
| GOTERM_MF_FAT | GO:0030246~carbohydrate binding | 12 | 12.24 | 5.87 | 0.001061551 | |
| GOTERM_BP_FAT | GO:0030334~regulation of cell migration | 7 | 7.14 | 7.37 | 0.027053907 | |
| GOTERM_CC_FAT | GO:0031012~extracellular matrix | 11 | 11.22 | 4.85 | 0.00382577 | |
| GOTERM_BP_FAT | GO:0035239~tube morphogenesis | 6 | 6.12 | 8.41 | 0.038030794 | |
| GOTERM_BP_FAT | GO:0040012~regulation of locomotion | 8 | 8.16 | 7.42 | 0.009678498 | |
| GOTERM_CC_FAT | GO:0044420~extracellular matrix part | 6 | 6.12 | 7.80 | 0.02412341 | |
| GOTERM_CC_FAT | GO:0044421~extracellular region part | 20 | 20.41 | 3.17 | 0.001390667 | |
| GOTERM_BP_FAT | GO:0048514~blood vessel morphogenesis | 10 | 10.20 | 8.44 | 0.000568267 | |
| GOTERM_BP_FAT | GO:0048754~branching morphogenesis of a tube | 6 | 6.12 | 16.43 | 0.003486756 | |
| GOTERM_BP_FAT | GO:0051270~regulation of cell motion | 7 | 7.14 | 6.46 | 0.036280406 | |
| GOTERM_BP_FAT | GO:0070482~response to oxygen levels | 9 | 9.18 | 11.36 | 0.0005119 | |
| KEGG_PATHWAY | hsa04510:Focal adhesion | 8 | 8.16 | 5.33 | 0.023639224 | |
| REACTOME_PATHWAY | REACT_12529:Signaling by VEGF | 3 | 3.06 | 35.64 | 0.020404263 | |
| REACTOME_PATHWAY | REACT_604:Hemostasis | 8 | 8.16 | 4.45 | 0.016837454 | |

| **Table S7. Enriched GO terms and pathways for DEGs down-regulated in the iron deficient group** | | | | | |
| --- | --- | --- | --- | --- | --- |
| **Category** | **Term** | **Count** | **%** | **Fold Enrichment** | **Benjamini** |
| GOTERM_CC_FAT | GO:0044421~extracellular region part | 12 | 23.08 | 3.99 | 0.009063746 |
| GOTERM_CC_FAT | GO:0005576~extracellular region | 16 | 30.77 | 2.54 | 0.023185887 |
| GOTERM_CC_FAT | GO:0005615~extracellular space | 9 | 17.31 | 4.20 | 0.028559845 |
| KEGG_PATHWAY | hsa04360:Axon guidance | 5 | 9.62 | 8.96 | 0.041457329 |

**Figure S1. Evidence of iron deficiency and reduced cognition in the iron deficient pigs.**

**
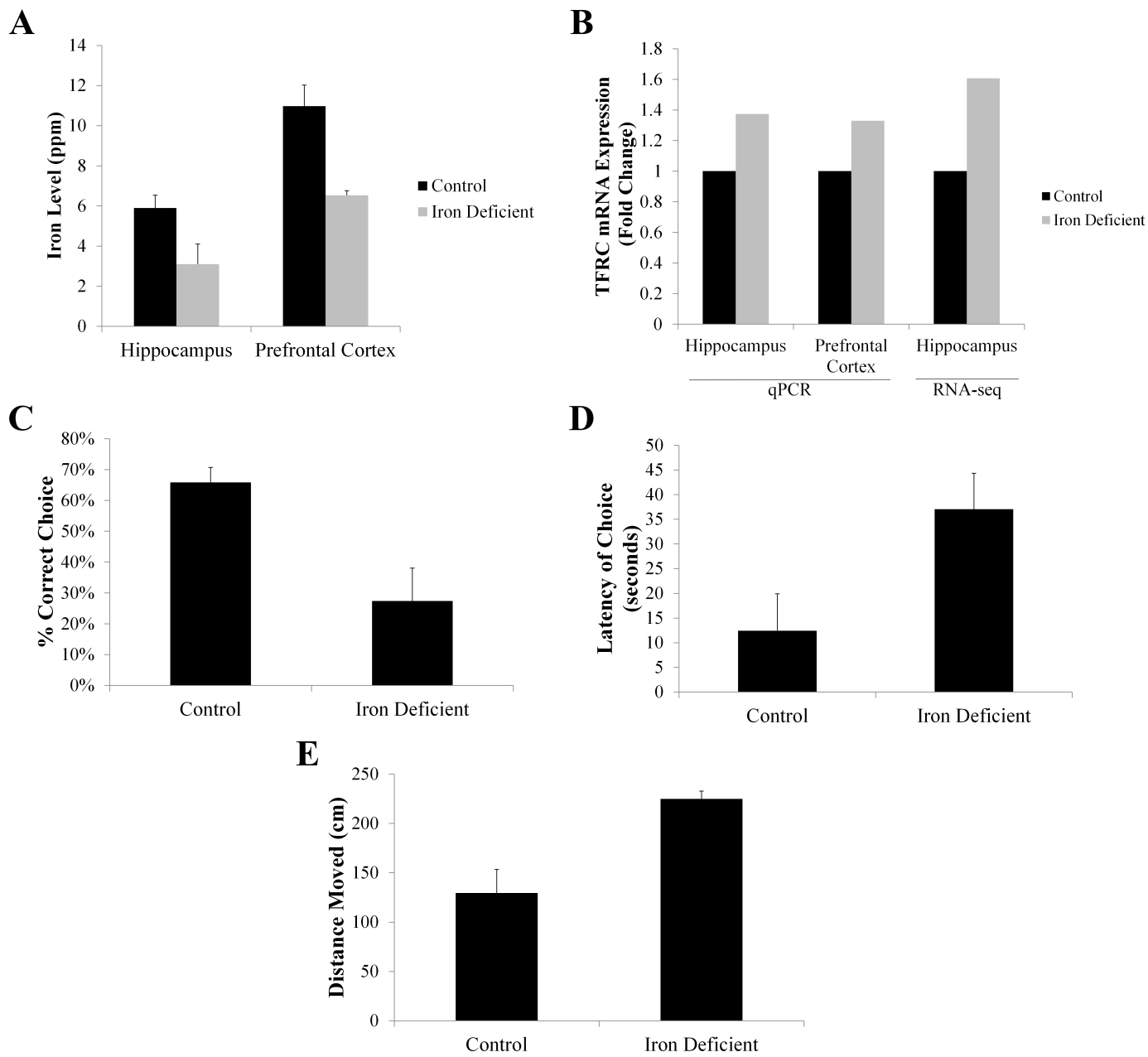
**

A. Average iron levels in the hippocampus and prefrontal cortex in parts per million (ppm). B. Average *TFRC* expression in the hippocampus and prefrontal cortex, represented as the fold change in expression relative to the control pigs. C. Average proportion of correct choices made during testing in a cognitive, spatial, T-maze task. D. Average latencies to make a reward arm choice, and E. average distance moved in the maze. Error bars denote standard error.

**Figure S2. Correlation analysis of CpG methylation levels.**


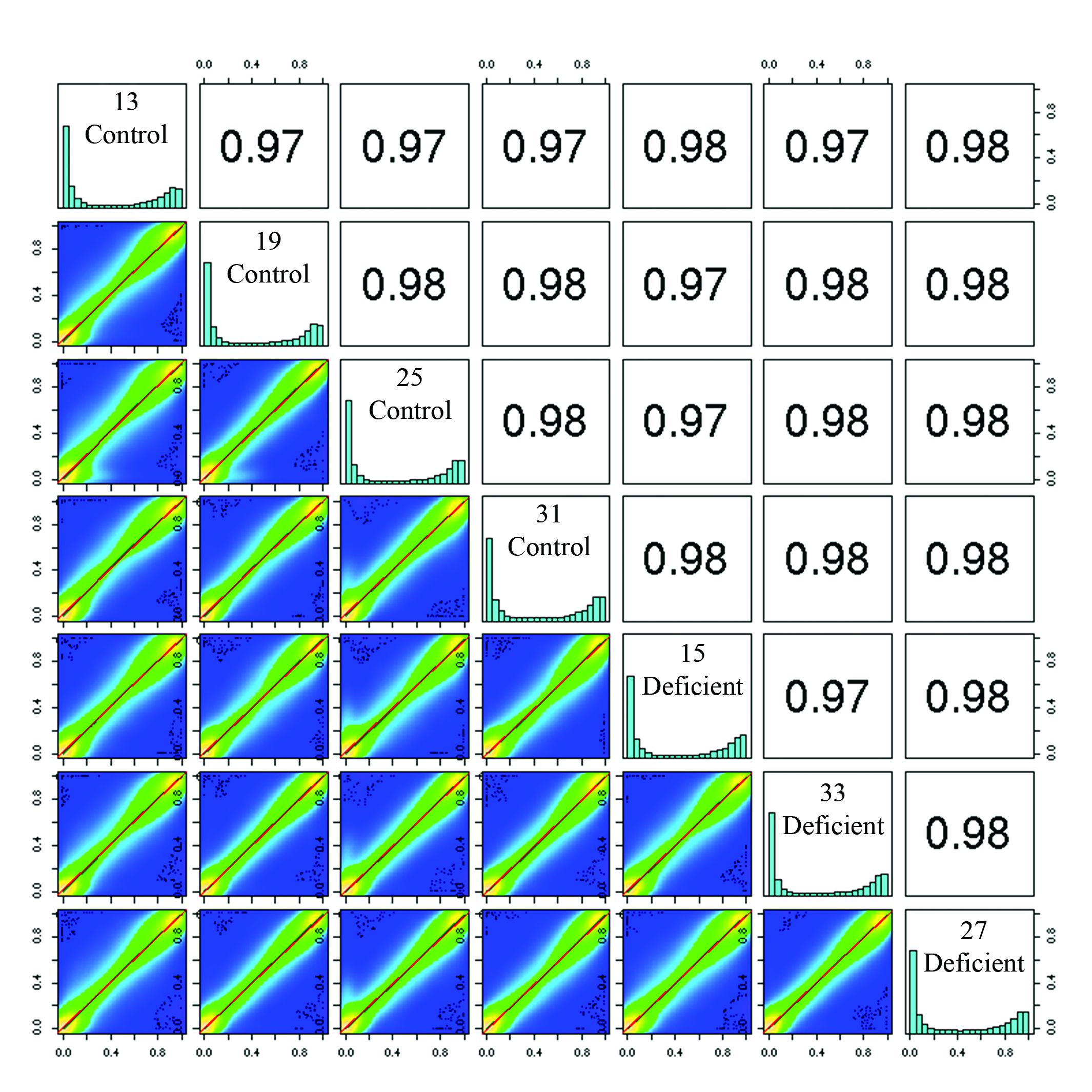


Density plots, histograms, and Pearson’s correlation coefficients for all samples based on the methylation level of all covered CpG sites.

**Figure S3. Correlation analysis of non-CpG methylation levels.**


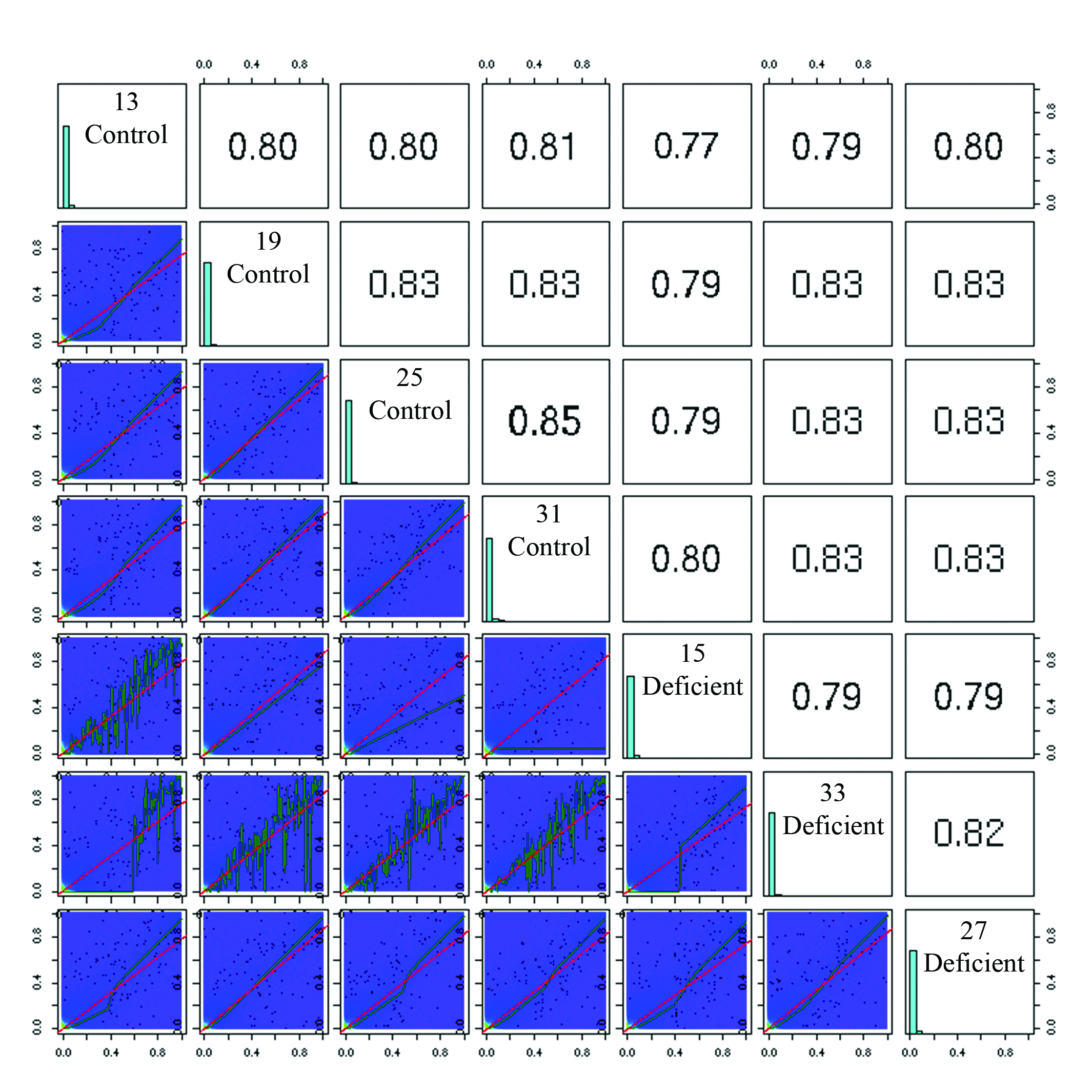


Density plots, histograms, and Pearson’s correlation coefficients for all samples based on the methylation level of all covered non-CpG sites.
